# The fitness cost of mis-splicing is the main determinant of alternative splicing patterns

**DOI:** 10.1186/s13059-017-1344-6

**Published:** 2017-10-30

**Authors:** Baptiste Saudemont, Alexandra Popa, Joanna L. Parmley, Vincent Rocher, Corinne Blugeon, Anamaria Necsulea, Eric Meyer, Laurent Duret

**Affiliations:** 1 Institut de Biologie de l’Ecole Normale Supérieure (IBENS), CNRS, Inserm, Ecole Normale Supérieure, PSL Research University, F-75005 Paris, France; 2 (Epi)genomics of Animal Development Unit, Department of Developmental and Stem Cell Biology, Institut Pasteur, 75015 Paris, France; 3 Université de Lyon, Université Claude Bernard, CNRS, Laboratoire de Biométrie et Biologie Evolutive UMR 5558, F-69100 Villeurbanne, France; 4 CeMM Research Center for Molecular Medicine of the Austrian Academy of Sciences, Lazarettgasse 14 AKH BT25.3, 1090 Vienna, Austria; 5 General Bioinformatics, Reading Enterprise Centre, The University of Reading, Whiteknights Road, Reading, RG6 6BU UK

**Keywords:** Alternative splicing, Random genetic drift, Selectionist/neutralist debate

## Abstract

**Background:**

Most eukaryotic genes are subject to alternative splicing (AS), which may contribute to the production of protein variants or to the regulation of gene expression via nonsense-mediated messenger RNA (mRNA) decay (NMD). However, a fraction of splice variants might correspond to spurious transcripts and the question of the relative proportion of splicing errors to functional splice variants remains highly debated.

**Results:**

We propose a test to quantify the fraction of AS events corresponding to errors. This test is based on the fact that the fitness cost of splicing errors increases with the number of introns in a gene and with expression level. We analyzed the transcriptome of the intron-rich eukaryote *Paramecium tetraurelia*. We show that in both normal and in NMD-deficient cells, AS rates strongly decrease with increasing expression level and with increasing number of introns. This relationship is observed for AS events that are detectable by NMD as well as for those that are not, which invalidates the hypothesis of a link with the regulation of gene expression. Our results show that in genes with a median expression level, 92–98% of observed splice variants correspond to errors. We observed the same patterns in human transcriptomes and we further show that AS rates correlate with the fitness cost of splicing errors.

**Conclusions:**

These observations indicate that genes under weaker selective pressure accumulate more maladaptive substitutions and are more prone to splicing errors. Thus, to a large extent, patterns of gene expression variants simply reflect the balance between selection, mutation, and drift.

**Electronic supplementary material:**

The online version of this article (doi:10.1186/s13059-017-1344-6) contains supplementary material, which is available to authorized users.

## Background

The maturation of a primary transcript by the spliceosome can lead to the production of diverse transcripts, via the use of different splice sites and/or intron retention (IR). Alternative splicing (AS) is widespread in eukaryotes and it has been postulated that it might considerably expand the functional repertoire of eukaryotic genomes [1–3]. Many case studies have shown that some AS events are functional, i.e. that they play a physiological role, beneficial for the fitness of the organism (for review, see [4]). However, like any biological machinery, the spliceosome is not 100% accurate and the splicing of primary transcripts occasionally leads to the production of spurious messenger RNAs (mRNAs). These erroneous transcripts represent a waste of resources and may lead to the production of toxic protein variants and hence are expected to be deleterious for the fitness of organisms. Indeed, several quality control mechanisms exist in eukaryotic cells to mitigate the negative impact of erroneous transcripts [5]. In particular, the nonsense-mediated decay (NMD) machinery is able to recognize and degrade cytoplasmic transcripts containing premature termination codons (PTCs) [6]. However, these quality-control processes themselves are not 100% efficient. Hence, any transcriptome necessarily includes a fraction of variants that correspond to splicing errors and their frequency relative to functional AS events remains open for debate.

In a large majority of cases, splice variants contain PTCs (i.e. encode truncated proteins) and only a very small fraction (<0.6%) of annotated AS events lead to the production of a detectable amount of protein [7]. The subset of AS variants that are detected in proteomic studies shows clear signs of protein functionality: 96% of them maintain the reading frame [8]; they rarely disrupt protein domains [7, 9]; and they are highly conserved, from mammals to bony fish [7]. This contrasts with the bulk of AS events detected within transcriptomes: 58% of them induce frameshifts [10] and 70% disrupt protein domains [7]. Moreover, comparative transcriptomic analyses revealed that only 1–3% of exon-skipping events detected by RNA-sequencing (RNA-seq) are conserved beyond mammals [11, 12] and alternative splice sites show no sign of selective constraint [10]. The subset of exon-skipping events that are strongly tissue-specific and that preserve the reading frame is generally more conserved, which clearly suggests that this subset includes some functional events [11–14]. However, these cases represent only a small fraction of all AS events [11–14]. These observations indicate that only a small minority of AS events are involved in the production of functional protein variants (for review, see [15]). This led some authors to conclude that the vast majority of AS events correspond to splicing errors [10, 16–18] (we will hereafter refer to this hypothesis as the “noisy splicing” model).

However, this interpretation is contested by other authors who argue that AS might play another important role, not linked to the production of functional protein variants, but to the regulation of gene expression. Indeed, the maturation of primary transcripts into PTC-containing splice variants, which then get degraded by NMD, can be used as a way to regulate the amount of mRNA available for protein production (this post-transcriptional regulation pathway is termed AS-NMD, for AS coupled with NMD; for review, see [19, 20]). AS-NMD notably plays an important role in the regulation of genes involved in the splicing process itself, presumably to maintain the homeostasis of splicing factors via auto-regulatory loops [21, 22]. Interestingly, although the regulation of splicing factors by AS-NMD is well conserved across animals, the AS events that trigger NMD in these genes often involve different splice sites [23]. The rapid evolution of AS events in mammals is therefore not necessarily in contradiction with the hypothesis that many of them play an important regulatory role. The comparison of transcriptomes in normal vs NMD-deficient cells revealed that a large fraction of genes produce splice variants (in a broad sense, i.e. including cases of IR) that are targeted by NMD [18, 24–27]. This pattern is widespread in eukaryotes and is not restricted to genes encoding splicing factors. Importantly, patterns of AS vary among tissues and during cell differentiation [28–30]. This led several authors to propose that AS-NMD might play a critical role in broadly regulating expression of a large percentage of genes [28–33].

Beyond a few case studies that provided clear evidence of genes regulated by AS-NMD, we still lack a global picture of the relative prevalence of functional AS compared to splicing errors. We propose here a test to quantify the fraction of splice variants corresponding to errors, i.e. having a negative impact on the fitness of organisms. The basis of this test is that the strength of splice signals is expected to reflect a balance between selection (which favors alleles that are optimal for splicing efficiency) and mutation and random genetic drift (which can lead to the fixation of non-optimal alleles) [34]. This selection-mutation-drift equilibrium therefore predicts a higher splicing accuracy at introns where errors are more deleterious for the fitness of organisms. Hence, if AS events predominantly correspond to splicing errors, one should expect a negative correlation between the rate of AS events and their cost in terms of resource allocation (metabolic cost, mobilization of cellular machineries). The noisy splicing model therefore makes several specific predictions regarding the AS rate according to whether splice variants are detectable by NMD and according to the expression level, length, and number of introns of genes.

**Fig. 1:**
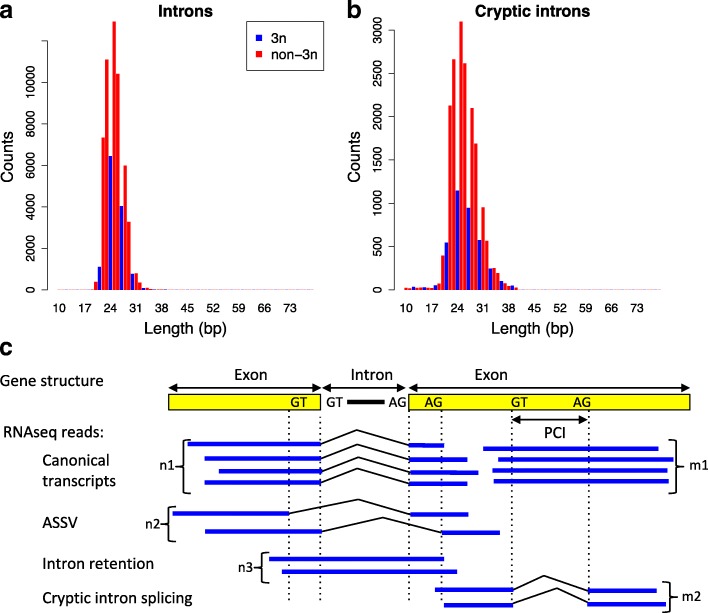
Introns and cryptic introns in *P. tetraurelia*. **a** Length distribution of introns (n = 65,159). **b** Length distribution of cryptic introns (n = 20,719 cryptic introns detected in wild-type or NMD-deficient cells). Introns and cryptic introns of length multiple of three (3n) or non-multiple of three (non-3n) are displayed in *blue* and *red*, respectively. **c** Quantification of splicing variation. For each intron, we identified all RNA-seq reads spanning both flanking exons and counted the number of reads corresponding to the canonical transcript (n1), to usage of 5′ or 3′ alternative splice sites (ASSV, n2), and to IR (n3). The IR rate is defined as n3/(n1 + n2 + n3), the ASSV rate is n2/(n1 + n2 + n3). Similarly, for potential cryptic introns (PCIs), the splice rate is defined as m2/(m1 + m2)

We first implemented this test in the ciliate *Paramecium tetraurelia*. The intron density in this organism (2.3 introns per gene on average) is similar to that observed in many other unicellular eukaryotes, and some animals, such as drosophila [35]. One major advantage of this organism is that its introns are very short (25.1 bp on average, with 99.9% of them in the range of 20–35 bp; Fig. 1a), i.e. much shorter than RNA-seq sequence reads, which greatly simplifies the detection and classification of AS events. In particular, cases of IR can be identified directly by detecting sequence reads spanning the entire intron and its flanking exon boundaries (Fig. 1c). Moreover, given its high number of genes (~40,000), this genome allows the analysis of a large dataset of introns (>90,000 introns). Finally, this organism already proved to be a good model to reveal important general features of splicing control in eukaryotes [36]. Here we present a comprehensive characterization of AS in the transcriptomes of normal and NMD-deficient paramecia to test the AS-NMD and noisy splicing models. We then ran the same test using previously published human transcriptome datasets and we quantified the fitness cost of mis-splicing in humans by analyzing polymorphism data. Our analyses reveal that the vast majority of splice variants correspond to errors.

## Results and discussion

### Quantification of splicing variants in Paramecium

For a given gene, the abundance of splicing variants depends both on the intrinsic strength of splicing signals and on the relative stability of the different variants. Thus, to study the determinants of alternative splicing in *P. tetraurelia,* we sequenced the polyadenylated RNA fraction of cells, either in normal state (hereafter denoted wild-type [WT]) or rendered NMD-deficient by knocking down one of the main components of the NMD machinery (Upf1, Upf2, or Upf3). The inactivation of Upf genes leads to stabilization of PTC-containing transcripts that would normally be degraded by the NMD machinery, thus providing a proxy for the intrinsic splicing efficiency of introns.

We generated ten RNA-seq datasets (Additional file 1: Table S1): six distinct NMD knockdown experiments and four replicates of WT cell cultures (see “Methods”). All biological replicates gave similar results (Additional file 1: Figures S1 and S2). We therefore pooled the sequencing datasets, to increase the per gene read depth (50% of genes have a read depth > 41 and > 85 in WT and in NMD-deficient samples, respectively). We detected splicing events by mapping sequence reads to the genome. These splicing events were then compared to gene models of the reference genome annotation, which includes 39,642 protein-coding genes, among which 31,632 contain introns (n = 90,287 introns) [37].

We detected three types of AS events (Fig. 1c): IR; alternative splice site variants (ASSV); and splicing of cryptic introns (i.e. introns with both splice sites located within an annotated coding exon). It is important to note that the classification of splice variants relies on the definition of a canonical form (Fig. 1c): the distinction between a “cryptic intron” and a “retained intron” depends on which variant is considered as the reference. For the vast majority of introns (97.8%), we observed one single major splice form, at least five times more abundant than other forms (Additional file 1: Figure S3). We therefore decided to define the canonical form as the one that is the most abundant in WT cells (see Additional file 1: Text S1). To be able to identify canonical forms, we restricted all subsequent analyses to genomic segments covered by at least ten RNA-seq reads in WT samples. This subset includes 65,159 annotated introns (which constitute our reference intron dataset).

To compare AS rates between different samples, it is necessary to normalize variant counts by the sequencing depth [38]. For introns, we computed the rates of retention and ASSV, defined as the proportion of variant reads among all reads spanning these reference introns (Fig. 1c). For cryptic introns, we considered all DNA segments potentially subject to cryptic splicing, i.e. segments of length 20–35 nt (matching the size distribution of observed introns and cryptic introns, Fig. 1), entirely located within an exon, starting with GT and ending with AG. These segments will hereafter be referred to as potential cryptic introns (PCIs). The rate of cryptic intron splicing is defined by the proportion of spliced reads among all reads spanning PCIs (Fig. 1c).

The average IR rate is about five times higher than the ASSV rate and 100 times higher than the splice rate of PCIs (Table 1). However, given the very large number of PCIs (on average there are 34.9 PCIs per gene vs only 2.3 introns), cryptic introns constitute a substantial fraction (6.9%) of all splice variants. Overall, combining all samples (WT and NMD-deficient), 95.0% of intron-containing genes show evidence of splicing variability in at least one of their introns and 32.3% of genes contain at least one detected cryptic intron (Additional file 1: Table S2). IR and ASSV rates are comparable to those observed in humans (Table 1). We did not observe any case of exon skipping in paramecia, but we detected 20,719 cryptic introns, 20 times more than reported in *Arabidopsis thaliana* and in humans [39]. This probably reflects the fact that the splicing machinery of paramecium only recognizes very short introns, which increases the risk of excising cryptic introns within exons, but precludes exon skipping.

**Table 1:** Summary of AS rates in paramecia and human

	*P. tetraurelia*	Human
Number of protein-coding genes	39,642	19,919
Mean (median) number of introns per gene	2.3 (2.0)	9.3 (7.0)
Average ASSV rate per intron	0.6%	1.9%
Average IR rate per intron	3.3%	3.4%
Mean (median) number of PCIs per gene	34.9 (26)	NA
Average splice rate per PCI	0.026%	NA

### Impact of NMD on steady-state levels of splice variants

**Fig. 2:**
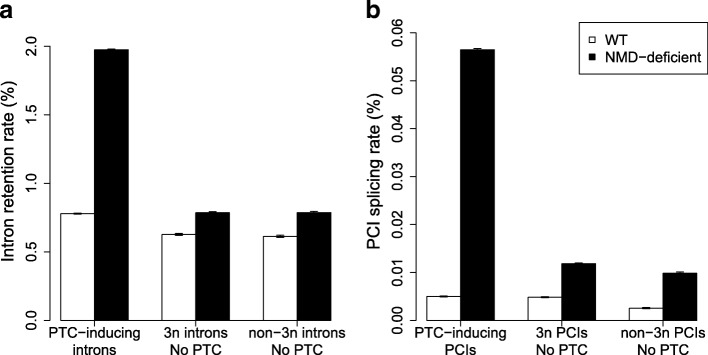
Impact of NMD on observed AS rates. AS events (IR or cryptic intron splicing) are classified into three groups according to their NMD-visibility: PTC-inducing events (i.e. NMD-visible); events that do not introduce frameshift or PTC (3n no PTC); events that create a frameshift but without introducing a PTC (non-3n no PTC). The two latter categories are not detectable by NMD. AS rates in WT and in NMD-deficient cells were computed globally within each bin, as the proportion of AS reads among all reads spanning introns (or PCIs) from that bin. *Error bars* represent the 95% confidence interval (CI) of this proportion. **a** IR (n = 65,159 introns). **b** Splicing of PCIs (n = 1,383,067 PCIs)

We classified splice variants in three categories according to their impact on the translation reading frame: (1) PTC-inducing variants; (2) variants that do not introduce frameshift or PTC (3n no PTC); (3) variants that induce a frameshift but without introducing a PTC (non-3n no PTC). Variants from the first category are NMD-visible, whereas the other two are not detectable by NMD. Among all PCIs, 63.8% are predicted to lead to NMD-visible transcripts in case of splicing, while 80.1% of introns are predicted to be NMD-visible in case of retention. As expected, the abundance of NMD-visible variants is strongly increased in NMD-deficient cells compared to WT cells (Fig. 2). For NMD-invisible variants, we observed a weak but significant increase in NMD-deficient cells compared to WT cells (Fig. 2). This increase probably reflects an indirect consequence of NMD inactivation: in many species, genes encoding splicing factors are regulated by AS-NMD [21, 22] and we observed the same pattern in paramecia (Additional file 1: Text S2, Additional file 1: Figure S4). Hence, the inactivation of NMD is expected to alter the efficiency of the splicing machinery, and thereby to indirectly affect the overall splicing pattern. The variation in AS rate for NMD-invisible variants is, however, much weaker than that observed for NMD-visible variants, which indicates that NMD directly affects the steady state levels of PTC-containing splice variants.

### Lower rate of alternative splicing in long and highly expressed genes

**Fig. 3:**
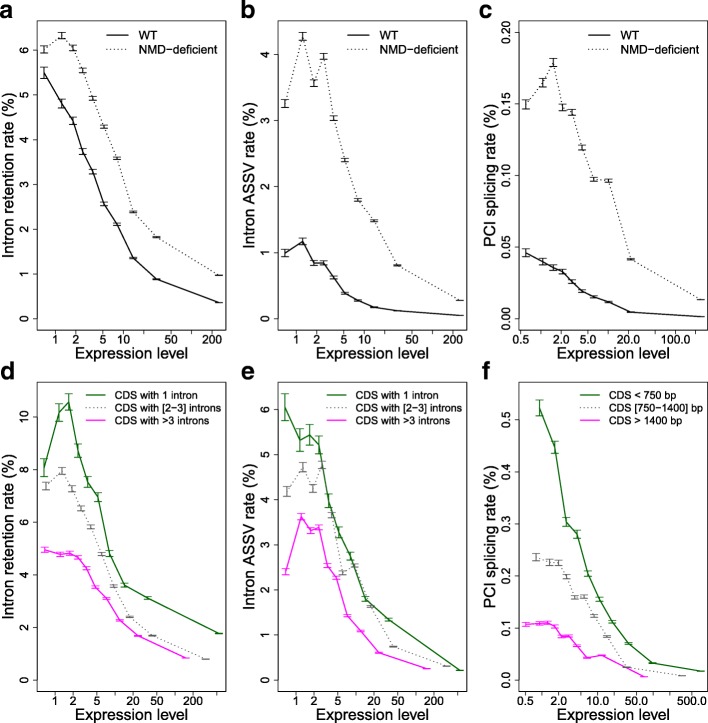
Relationship between AS rate and gene features: expression level, number of introns, or length of coding regions. Introns (n = 65,159) and PCIs (n = 1,383,067) were classified into ten bins of equal sample size, according to gene expression levels in WT cells. The AS rate was computed globally within each bin, as the proportion of AS reads among all reads spanning introns (or PCIs) from that bin. *Error bars* represent the 95% CI of this proportion. **a** IR rate. **b** ASSV rate. **c** Rate of splicing at potential cryptic introns. **d**, **e** same as (**a**, **b**), but introns were first classified into three bins, according to the number of introns of the gene in which they are located: genes with 1 intron (n = 5606 introns), genes with 2–3 introns (n = 24,452 introns), genes with > 3 introns (n = 35,101 introns). **f** Same as (**c**), but PCIs were first classified into three bins, according to the length of the coding region (CDS) in which they are located: CDS < 750 bp (n = 169,030 PCIs), CDS 750–1400 bp (n = 406,460 PCIs), CDS > 1400 bp (n = 807,577 PCIs). **a**–**c** AS rates were measured in normal cells (WT, *black line*) and in NMD-deficient cells (*dashed line*). **d**–**f** AS rates were measured in NMD-deficient cells. Expression levels (RPKM) are represented in log scale

The previous observations indicate that AS-NMD might potentially contribute to the post-transcriptional regulation of many genes. However, they are also compatible with the hypothesis that most splice variants are errors and that NMD is used as a surveillance mechanism to degrade erroneous transcripts. This “noisy splicing” model makes several testable predictions, which are based on three points. First, the cost of splicing errors is expected to increase with gene expression level: for a given splicing error rate, the waste of resources (both in terms of metabolic cost and of futile mobilization of cellular machineries) will be larger for highly expressed genes, and hence, the selective pressure on splicing accuracy is expected to be stronger. In other words, if AS events predominantly correspond to errors, the selection-mutation-drift theory predicts that the AS rate should correlate negatively with gene expression level. To test this prediction, we classified introns (or PCIs) into ten bins of equal sample size according to their gene expression level and computed the AS rate within each bin. In agreement with the “noisy splicing” model, we observed a strong decrease in AS rate with increasing expression level, for IR (Fig. 3a), ASSV (Fig. 3b), and cryptic intron splicing (Fig. 3c). This pattern is observed in both WT and NMD-deficient cells, which indicates that the observed variations reflect differences in intrinsic splicing efficiency.

The second point is that, for a given splicing error rate per intron, the rate of production of spurious transcripts increases with the number of introns present in a gene: the greater the number of introns, the greater the risk of having at least one error. The selective pressure on the strength of splice signals of each intron is therefore expected to increase with the number of introns in a gene and hence the AS rate (per intron) should be lower in genes with more introns. To test this prediction, we classified introns into three groups according to the number of introns present in their gene: genes with 1 intron; with 2–3 introns; and with at least 4 introns (mean = 5.2 introns) (the three groups correspond to 27.8%, 43.0%, and 29.2% of intron-containing genes, respectively). We then binned each group according to gene expression level and computed the AS rate per bin. Again, observations perfectly match predictions: for a given expression level, the AS rate per intron is higher in genes with fewer introns, both for IR (Fig. 3d) and for ASSV (Fig. 3e).

The third point is that the risk of cryptic intron splicing increases with the number of PCIs and therefore with the length of coding sequences (CDSs). The selective pressure to limit the strength of cryptic splice signals should therefore increase with CDS length and PCIs in long CDSs should have a lower splicing rate compared to PCIs in short CDSs. To test this prediction, we classified PCIs into three groups according to the length of the CDS in which they are located (each group corresponds to one-third of all genes) and then binned each group by gene expression level and computed the PCI splicing rate per bin. Again, the predictions of the model fit the observations: for a given expression level, the splicing rate per PCI is lower in genes with longer CDSs (Fig. 3f). Thus, all observations fit the three predictions of the “noisy splicing” model.

### The genome-wide AS pattern is dominated by splicing errors

The previous results indicate that the level of constraints against splicing errors is maximal in highly expressed genes containing many introns and/or encoding long CDSs (Fig. 3) (we will hereafter refer to this class of long/intron-rich highly expressed genes as “highly constrained” genes). The strong relationship between AS rate and expression level can be used to quantify the splicing error rate in each bin of expression. The proportion of AS events that correspond to splice errors (*P* ^*e*^) is given by:

1 $$ {P}^e=\frac{A{S}^e}{A{S}^e+A{S}^f} $$

where *AS* ^*f*^ is the rate of functional AS events and *AS* ^*e*^ is the rate of erroneous splicing.

The ratio of the AS rate in a given bin of expression (*i*) over the AS rate in highly constrained genes (*h*) is given by:

2 $$ {r}_i=\frac{A{S}_i}{A{S}_h}=\frac{A{S}_i^e+A{S}_i^f}{A{S}_h^e+A{S}_h^f} $$

Under the assumption that the rate of functional AS events is the same for both gene classes (*AS*_*h*_^*f*^ *= AS*_*i*_^*f*^ *= AS* ^*f*^ ; see Additional file 1: Text S4 for a discussion about this assumption), the proportion of splicing errors in expression bin (*i*) can be written as:

3 $$ {P}_i^e=1-\frac{A{S}^f}{r_i\left(A{S}_h^e+A{S}^f\right)} $$

If selection is very strong in the set of highly constrained genes, so that the splicing error rate is negligible compared to the rate of functional AS events in that gene set (i.e. ${AS}_h^e\ll {AS}^f$), then Eq. 3 simplifies to:

4 $$ {P}_i^e=1-\frac{1}{r_i} $$

As a reference for highly constrained genes, we considered genes with a high expression level (top 10%) and with > 3 introns (for the quantification of erroneous ASSV and IR events) or with a CDS > 1400 bp (for the quantification of erroneous cryptic intron splicing). In WT cells, we observed that the ratio of the AS rate in genes with median expression level over the AS rate in highly constrained genes are *r*_*i*_ = 12.0, *r*_*i*_ = 20.3, and *r*_*i*_ = 49.3 for IR, ASSV, and cryptic intron splicing, respectively. According to Eq. 4, this implies that for a median gene, 92–98% of splice variants detected in WT cells result from errors and this proportion might even be higher if the splicing error rate in highly expressed genes is not negligible (Eq. 3).

These estimates are based on the assumption that, on average, the rate of functional AS does not vary with gene expression level (i.e. *AS* ^*h*^_*f*_ *= AS* ^*l*^_*f*_ *= AS*_*f*_ in Eq. 3). One may argue, however, that variation in AS rate with expression level might reflect differences in the propensity to use AS-NMD: it is in principle possible that weakly expressed genes are more prone to use AS-NMD to fine-tune their expression level (i.e. *AS* ^*l*^_*f*_ > *AS* ^*h*^_*f*_). For instance, one might speculate that highly expressed genes are preferentially regulated at the transcriptional level, to avoid the waste of resources caused by the post-transcriptional AS-NMD pathway. Furthermore, if gene regulation via AS-NMD requires only one AS-prone intron per gene, then this could explain why the average AS rates (measured over all introns) decrease with increasing number of introns per gene (Fig. 3). Thus, although all previous observations are consistent with the predictions of the noisy splicing model, they do not formally invalidate the AS-NMD hypothesis.

**Fig. 4:**
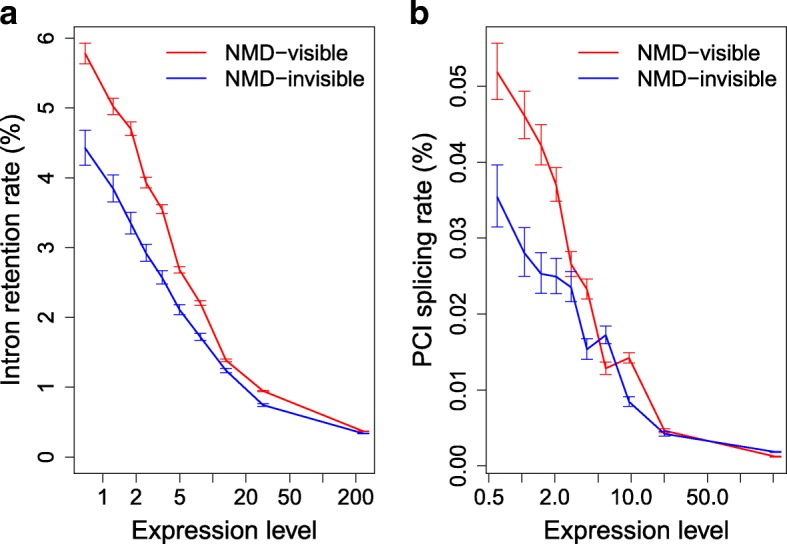
Relationship between AS rate and expression level, for NMD-visible or NMD-invisible AS events. **a** Introns were first classified into two groups according to their NMD-visibility in case of retention events (n = 52,163 NMD-visible introns, in *red*, and n = 12,996 NMD-invisible introns, in *blue*), and then further grouped into ten bins of equal sample size, according to gene expression levels in WT cells. IR rates (in WT cells) were measured globally in each bin. *Error bars* represent the 95% CI of the proportion of AS reads. **b** Same as (**a**), but for the splicing of PCIs: n = 882,579 NMD-visible PCIs and n = 500,488 NMD-invisible PCIs. Expression levels (RPKM) are represented in log scale

One important point to note, however, is that AS events that do not introduce a PTC cannot contribute to gene regulation via AS-NMD. Hence, if the correlation between AS rate and expression level was due to a higher propensity of lowly expressed genes to be regulated by AS-NMD, then this correlation should be observed exclusively for AS events that can trigger NMD. To test this prediction, we analyzed splicing variants according to their NMD-visibility. We observed a strong negative relationship between AS rate and gene expression level, both for NMD-visible and NMD-invisible splicing variants (Fig. 4 for WT cells and Additional file 1: Figure S5 for NMD-deficient cells). In other words, weakly expressed genes show a high rate of alternative splicing events, even for NMD-invisible splicing events, which, by definition, cannot contribute to the regulation of gene expression by AS-NMD. Thus, the observed relationships between gene expression level and AS rates (NMD-visible or not) provide strong evidence against the AS-NMD model. The most parsimonious explanation is that the excess of AS in weakly expressed genes compared to highly expressed genes simply reflects differences in the selection-mutation-drift equilibrium: these genes are under weaker selective pressure for splicing accuracy and hence show a higher rate of splicing error. If this interpretation is correct, then our calculations imply that for a median gene, at least 92–98% of splice variants detected in WT cells correspond to weakly deleterious errors.

### A dual strategy to limit the cost of splicing errors

In NMD-deficient cells, the IR rate is much higher for NMD-visible introns than for NMD-invisible introns, which indicates that the former has a lower intrinsic splicing efficiency (Fig. 2a). The difference in intrinsic splicing efficiency results, at least in part, from a difference in the strength of splice signals: on average, 77.4% of NMD-invisible introns match the consensus splicing signals [GTA..TAG], compared to only 69.8% for NMD-visible introns (Chi-squared test = 289.1, *p* < 10^–15^). However, in WT cells, the observed AS rate is similar for both categories of introns. This implies that the efficacy of NMD to eliminate transcripts with retained introns is strong enough to compensate the lower intrinsic splicing efficiency of NMD-visible introns.

The same pattern is observed for PCIs: in WT cells, NMD-visible and NMD-invisible PCIs show similar rates of splicing (Fig. 2b, Additional file 1: Figure S6A), despite the fact that the intrinsic rate of splicing of PCIs (observed in NMD-deficient cells) is about five times higher for NMD-invisible compared to NMD-visible PCIs (Fig. 2b, Additional file 1: Figure S6B). Thus, again, the higher intrinsic propensity of NMD-visible PCIs to be spliced out is compensated by the activity of NMD in WT cells.

### Patterns of alternative splicing in humans are consistent with the noisy splicing model

**Fig. 5:**
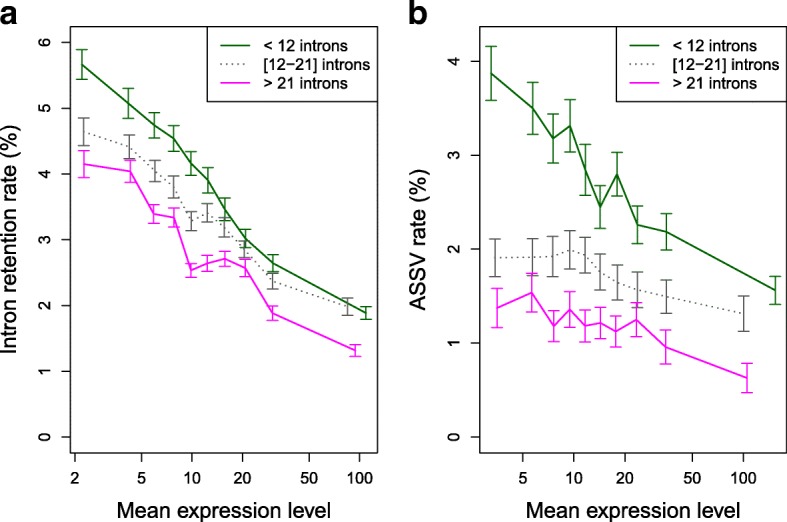
Relationship between AS rate, expression level, and number of introns in human genes. **a** IR rate (n = 118,703 introns). **b** ASSV rate (n = 102,697 introns). In both panels, introns were first classified into three groups of equal sample size, according to the number of introns of the genes in which they are located (genes with < 12 introns, genes with 12–21 introns, genes with > 21 introns), and then further grouped into ten bins of equal sample size, according to gene expression levels. We computed the average AS rate (IR or ASSV) over all introns within each bin. *Error bars* represent the 95% CI of the mean. Expression levels (RPKM, averaged over the 52 samples) are represented in log scale

To test whether the observations that we made in a unicellular organism (*P. tetraurelia*) hold true in multicellular eukaryotes, we quantified ASSV in human introns, using previously published RNA-seq datasets coming from 25 different tissues or cell types (Additional file 1: Table S3). Note that the ASSV events that we detected in humans include not only alternative 3′ or 5′ splice site usage (as in paramecia, Fig. 1c), but also exon skipping, alternative initial/terminal exons, or mutually exclusive exons [40]. We also re-analyzed a dataset published by Braunschweig et al. [29], which provides a quantification of IR rates of human introns in 52 different tissues and cell types. In agreement with previous reports [29], we observed that the IR rate (averaged over the 52 samples) decreases with increasing gene expression level. According to the authors, this observation supports their conclusion that gene expression is regulated through NMD acting on transcripts with retained introns [29]. However, the negative relationship between IR rate and expression level is observed both for NMD-visible events and for NMD-invisible events (Additional file 1: Figure S7A), which is not consistent with the AS-NMD model. Moreover, we observed that for a given expression level, the IR rate (per intron) decreases with increasing number of introns in the gene (Fig. 5a). We observed exactly the same patterns for ASSV rates (Fig. 5b and Additional file 1: Figure S7B). Thus, in humans as in paramecia, variations in ASSV and IR rates fit with the predictions of the noisy splicing model.

As a reference dataset of highly constrained human genes, we considered genes with a high expression level (top 10%) and with > 21 introns (top 33%). The ratio of the AS rate in genes with median expression level over the AS rate in highly constrained genes are *r*_*i*_ = 3.1 and *r*_*i*_ = 3.6 for IR and ASSV, respectively. According to Eq. 4, this implies that for median genes, at least 68% of IR events and 72% of ASSV events correspond to errors. These estimates are lower than in paramecium (92% for IR, 95% for ASSV), which might reflect a higher proportion of functional AS events in mammals than in ciliates. One noticeable difference between AS patterns in these organisms is that exon-skipping is common in mammals, but absent in paramecium. Interestingly, in mammals, exon-skipping events that preserve the reading frame are more conserved than other AS events, which indicates that this subset includes a higher fraction of functional events [14]. However, it should be noted that this subset represents only ~ 15% of ASSV events in human [14]. In fact, the difference between human and paramecium estimates might simply result from a limitation of our methodology. Indeed, these estimates are based on the assumption that the error rate in the set of highly constrained genes is negligible. In paramecia, AS rates tend to plateau at high expression levels (Fig. 3), which is compatible with the hypothesis that this basal rate might correspond to functional splice variants. However, in human, contrary to paramecia, there is no sign that AS rates reach a basal value at high expression levels, both for IR and ASSV events (Fig. 5). It is therefore likely that the splicing error rate is substantial, even in the reference dataset of highly constrained genes. Hence, the above estimates are certainly an underestimate of the true splicing error rate in humans.

### Fitness impact of mis-splicing in humans

**Fig. 6:**
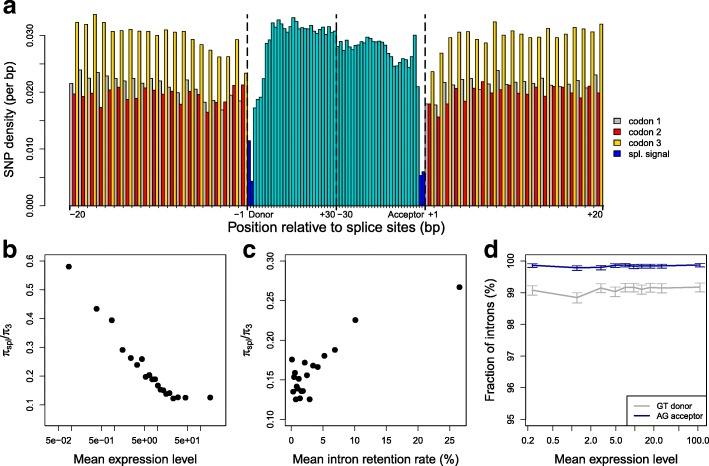
Variation in selective constraints on splice signals in human genes. **a** SNP density was measured in the vicinity of exon-intron boundaries (first and last 30 bp of introns and 20 bp of flanking exons), over all introns located between coding exons (n = 170,015). Splice sites (first and last 2 bp of introns) are displayed in *dark blue*, other intron positions in *light blue*. Within coding regions, the SNP density at each site was computed separately for the three codon positions (*gray*: position 1, *red*: position 2, *yellow*: position 3). **b** The level of selective constraints on splice signals increases with gene expression level. Introns were classified into bins of equal sample size, according to gene expression levels. Within each bin, the fitness impact of mutations on splice sites was estimated by measuring the ratio π_spl_/π_3_, where π_spl_ is the SNP density at splice sites and π_3_ is the SNP density at flanking third codon positions. **c** The level of selective constraints on splice signals decreases with increasing IR rate. Introns were classified into bins of equal sample size according to their average retention rate and the ratio π_spl_/π_3_ was measured in each bin. **d** The fraction of introns with consensus splice signals does not vary with gene expression level. The proportion of introns matching the consensus splice donor (GT) and the proportion of introns matching the consensus splice acceptor (AG) was computed for each bin of expression level. *Error bars* represent the 95% CI of this proportion. **b**, **d** Mean expression levels (RPKM) are represented in log scale

One strong assumption of the noisy splicing model is that the fitness impact of splicing errors increases with expression level. To test this hypothesis, we analyzed patterns of polymorphism in the vicinity of human splice sites. Splicing imposes strong constraints on donor and acceptor sites (defined as the first and last 2 nt of introns): 99.1% of human introns start with GT and 99.8% end with AG. As expected, these sites show evidence of strong purifying selection: the SNP density is 4.5-fold lower at splice sites than in flanking third codon positions (Fig. 6a). We quantified this selective pressure by measuring the ratio π_spl_/π_3_, where π_spl_ is the SNP density at splice sites and π_3_ is the SNP density at flanking third codon positions. We binned introns by gene expression level and computed this ratio in each bin. Interestingly, the π_spl_/π_3_ ratio is strongly correlated to gene expression level (R^2^ = 0.89, *p* < 10^–9^), with a fivefold difference between lowly and highly expressed gene sets (Fig. 6b). Note that contrarily to π_spl_, π_3_ does not correlate with gene expression level (Additional file 1: Figure S8), which confirms that variation in π_spl_/π_3_ reflects differences in the intensity of selection on splice sites. It should be stressed that the fraction of introns matching the GT..AG consensus does not vary with gene expression level (Fig. 6d). This implies that mutations occurring at donor and acceptor sites are ultimately counter-selected, even in weakly expressed genes. However, our observations (Fig. 6b) show that these mutations are more rapidly purged in highly expressed genes. This demonstrates that the fitness cost of mis-splicing increases with gene expression level.

To test whether IR rate co-varies with the fitness impact of mis-splicing, we binned introns according to their IR rate and computed π_spl_/π_3_ in each bin. We observed a positive correlation between π_spl_/π_3_ and the average IR rate per bin (R^2^ = 0.76, *p* < 10^–6^), with a twofold increase between bins of low IR compared to bins of high IR (Fig. 6c). Again, it is important to stress that the frequency of introns matching the GT..AG consensus does not vary with IR rate (Additional file 1: Figure S9). This implies that mis-splicing is deleterious, even in introns with high IR rate. However, in agreement with the noisy splicing model, introns that show a high IR rate correspond to introns where mis-splicing is relatively less deleterious.

## Conclusions

The efficiency of excision of introns by the spliceosome is affected by different signals, located within introns and flanking exons (splice sites, branch point, polypyrimidine tract, splicing enhancers, or silencers). Besides the two splice sites that are critical for the splicing reaction (almost always GT for the donor and AG for the acceptor), all other signals tolerate some sequence flexibility. The probability for a mutation affecting a splicing signal to reach fixation depends on its fitness impact (i.e. the selection coefficient, *s*) and on the power of random genetic drift (i.e. the effective population size, *N*_*e*_) [34]. There is therefore necessarily a limit to the point up to which selection can optimize the strength of splice signals: if the splicing error rate is already low, any mutation that further improves splicing efficiency will necessarily have a weak fitness impact and hence will be subject to random drift (the so-called drift barrier effect [41]). This drift barrier therefore determines a basal splicing error rate, which depends on the mutation rate, on *N*_*e*_, and on the fitness cost of splicing errors (*s*).

For a given error rate, errors are expected to be more costly (in terms of metabolic resources and mobilization of cellular machineries) in highly expressed genes. Hence the fitness cost of mis-splicing is expected to increase with increasing expression level. Indeed, this is precisely what we observed in humans: the strength of selection against deleterious mutations at splice sites is strongly correlated to gene expression level (Fig. 6b). Since the risk of producing erroneous transcripts increases with the number of introns, this implies that all else being equal, there should be a stronger selective pressure against mis-splicing in intron-rich genes. The mutation-selection-drift theory therefore predicts that introns from weakly expressed/intron-poor genes should accumulate more non-optimal substitutions in their splice signals and therefore should show a higher splicing error rate. The relationships that we observe between AS rate, expression level, and intron number are perfectly consistent with these predictions, both in human (Fig. 5) and in paramecia (Fig. 3).

There are two possible ways to limit the deleterious impact of erroneous splicing: (1) improve the strength of splicing signals to increase intrinsic splicing efficiency and avoid the use of cryptic signals (error prevention); or (2) ensure that transcripts are degraded by NMD in case of splicing error (error mitigation). We observed that both strategies are used: there is a deficit of introns and cryptic introns that cannot trigger NMD in case of splicing error; and the rare introns that are not NMD-visible show stronger splicing signals (Additional file 1: Text S3, Additional file 1: Figure S10). The analysis of AS rate in NMD-deficient cells shows that NMD-invisible introns have a much higher intrinsic splicing accuracy than NMD-visible ones. This difference demonstrates that the biophysical limits of splicing accuracy have not been reached and that it would be possible to further improve splicing accuracy of NMD-visible introns by genetic engineering. However, the mutation-selection-drift theory predicts that once the basal splicing error rate has been reached, by error prevention or by error mitigation, then selection cannot further improve splicing efficiency. Thus, this model predicts that the steady state level of erroneous transcripts (after quality control by NMD) should be the same for NMD-visible and NMD-invisible introns. And this is precisely what we observed: in WT cells, NMD-visible and NMD-invisible AS events show similar rates (Fig. 2).

The fitness cost of splicing errors depends on the frequency of transcripts subject to at least one erroneous splicing event. Owing to the short length of RNA-seq sequence reads, it is not possible to directly quantify AS rates per transcript. However, given that AS rates (per intron) are similar in human and in paramecia (Table 1) and that human genes contain on average 3–4 times more introns than paramecia, this implies that the frequency of transcripts subject to at least one erroneous splicing event must be much higher in human than in paramecia. This is consistent with the drift-barrier hypothesis, which predicts that humans should have a higher splicing error rate (per gene), owing to their larger mutational targets (more introns) and to their smaller effective population size [41, 42].

There is clear evidence that some AS events are functional [4]. Notably, we observed that AS-NMD probably plays an important role in the regulation of genes encoding splicing factors in paramecia (Additional file 1: Text S3), as previously shown in other eukaryotes [21, 22]. However, AS-NMD cannot explain the strong relationship between AS rate and expression level that is observed for NMD-invisible splicing variants (Fig. 4, Additional file 1: Figure S7). It has been recently shown that the retention of introns in nuclear transcripts (the so-called “detained” introns) might also contribute to the regulation of gene expression, independently of NMD [43]. If weakly expressed genes were more prone to use this regulatory pathway, this might explain the relationship observed between expression level and IR rate. However, this model does not explain the relationship between IR rate and intron number (Figs. 3d and 5a) and, most importantly, cannot explain the relationship between expression level and other classes of AS events (ASSV or cryptic intron splicing; Figs. 3 and 5). The most parsimonious explanation is that the excess of AS in weakly expressed/intron-poor genes results from the accumulation of maladaptive substitutions, driven by random genetic drift in genes where the selective pressure is weaker. Our observations indicate that for median genes, the vast majority of observed splice variants correspond to errors, in contradiction with the panglossian view of a widespread role of AS-NMD in fine-tuning the expression of genes. Of course, this does not negate the importance of AS-NMD in the regulation of some genes. However, our results highlight the necessity of a careful consideration of non-adaptive hypotheses before concluding about the functionality of AS events.

## Methods

### *Paramecium* strain, cell culture, and inactivation of NMD

The entirely homozygous strain 51 of *P. tetraurelia* was grown in a wheatgrass powder infusion medium bacterized with *Klebsiella pneumoniae* the day before use and supplemented with 0.8 mg.L^-1^ ß-sitosterol. NMD was inactivated either by RNAi-mediated silencing of UPF genes during vegetative growth of WT cells or by generating somatic knockouts, i.e. clones in which these genes are deleted from the macronucleus. RNAi treatment was based on the double-stranded RNA feeding technique [44]: briefly, cells were fed for seven days with *E. coli* (HT115) producing double-stranded RNA homologous to the target gene. Sequences used for silencing of UPF1A, UPF1B, UPF2, UPF3, and ICL7a (which encodes a cytoskeletal protein), were segments 1885–2289, 1887–2285, 1143–1546, 18–422, and 1–580 of the genes (from the ATG), respectively. These genes can be accessed with ParameciumDB (http://paramecium.cgm.cnrs-gif.fr/) under accession numbers GSPATG00034062001, GSPATG00037251001, GSPATG00017015001, GSPATG00001393001, and GSPATG00021610001, respectively. Somatic knockouts were generated by applying RNAi treatment during the development of a new somatic macronucleus, which results in the deletion of the targeted genes [45, 46]: WT conjugating pairs were transferred to “UPF” RNAi medium and, following their separation, individual exconjugants were isolated in the same medium. After 24 h of growth, cells were transferred to standard growth medium. Among the viable exconjugants obtained, somatic UPF deletions were screened for based on the slow growth phenotype and the inability to undergo autogamy, and later confirmed by Southern blots and PCR (Additional file 1: Figure S11).

### RNA-seq

Total RNA was extracted from cells grown on *K. pneumoniae* or the relevant feeding *E. coli* strains with the TRIzol (Invitrogen) procedure, modified by the addition of glass beads. All RNA samples were treated with DNase prior to library construction to minimize DNA contamination. For the first four RNA-seq datasets in Additional file 1: Table S1, poly(A) RNAs were purified from 100 μg of total RNA with the MicroPoly(A)purist kit (Ambion). Of the output, 25% was used for mRNA reverse transcription, using the SuperScript III kit (Invitrogen) and the anchor-oligo(dT) primer 5′-GCCCACCAGAGCCGGCGGATTTTTTTTTTTTTTTTT-3′. After alkaline lysis of RNA and removal of the oligo(dT) primer with G-50 columns (GE Healthcare), a poly(G) tail was added to single-stranded complementary DNAs (cDNAs) with terminal transferase (NEB) following the producer’s instructions. After phenol purification and ethanol precipitation, cDNAs were made double-stranded using the Phusion PCR enzyme (Finnzymes) and the anchor-oligo(dC) primer 5′-GCCCACCAGAGCCGGCGGACCCCCCCCCCCCCCCCC-3′. Double-stranded DNA was then purified using the Qiagen PCR purification kit and cDNA libraries were amplified by 15 cycles of PCR with the anchor primer. cDNA libraries were digested by EciI restriction enzyme (NEB) and purified (Qiagen) before addition of Illumina adaptors. For the last six RNA-seq datasets, library preparation and Illumina sequencing were performed at the ENS Genomic Platform (Paris, France). Poly(A) RNAs were purified from 1 μg of total RNA using oligo(dT). Libraries were prepared using the strand non-specific RNA-seq library preparation TruSeq RNA Sample Prep kit (Illumina) and multiplexed by 3 on 2 flowcell lanes. 101-bp paired-end read sequencing was performed on a HiSeq 1500 device (Illumina).

### Read mapping

The sequencing of these ten samples yielded a total of 40.8 Gb (from 247,653,027 fragments), 25.1 Gb from NMD-deficient cells, and 15.7 Gb from control cells (Additional file 1: Table S1). Reads were mapped against the *P. tetraurelia* reference genome assembly (accession number: CAAL01000000) [37], using TopHat (version 1.4.1) [47]. The minimal and maximal intron lengths were set to 10 nt and 500,000 nt, respectively. Reads that mapped at multiple positions on the genome were excluded from further analyses. Read coverage along transcription unit was obtained using annotated gene models from the reference genome [37]. The expression level of genes was measured in reads per kilobase per million mapped reads (RPKM).

### Detection of splicing events

For each annotated intron, we counted the number of mapped reads spanning both extremities (Fig. 1c). Reads aligning to the genome sequence without any gap were counted as IR. Reads showing a deletion corresponding exactly to the annotated intron were counted as splice events. Reads with a deletion that does not match the annotated intron (at one or both extremities) were counted as ASSV. Reads showing a deletion entirely located within an annotated coding exon were counted as cryptic intron splicing events (Fig. 1c).

The cell cultures that we analyzed are totally homozygous. However, it is important to note that in paramecia, the macronuclear genome is highly polyploid and that the different copies of a same gene may differ due to heterogeneity in the process of excision of internal eliminated sequences (IESs) [48]. Thus, a fraction of the diversity detected in the transcriptome may in fact result from this macronuclear genomic heterogeneity. Among all alignment gaps detected by TopHat, > 97% match the consensus intron boundaries (GT/AG), which indicates that most of them correspond to bona fide splice events. To avoid any confusion between splice variants and IES excision variants, we counted as splice variants only those matching the GT/AG consensus.

The classification of splice variants (IR, ASSV, or cryptic intron splicing) was based on the comparison with the canonical form, defined as the major form observed in WT cells. Among the 90,287 annotated introns, we selected those that are spanned by at least 10 reads in WT samples (n = 70,242). Among those ones, 4045 were never observed as spliced and 1038 correspond to minor splice forms. Thus, our reference dataset includes 65,159 introns (72% of the initial dataset).

### Quantification of AS rate

One important goal of this study was to analyze the relationship between AS rate and gene expression level. The AS rate at a given intron is defined by the proportion of splice variant reads among all reads spanning that intron (Fig. 1c). One difficulty is that the precision of this metric is strongly dependent on the sequencing read depth and, hence, the measure of the AS rate is much less accurate in weakly than in highly expressed genes. To circumvent this problem, we binned introns (or PCIs) by expression level and then measured the global AS rates in each bin (defined by the proportion of splice variant reads among all reads in that bin).

The measure of IR rate might potentially be biased by the presence of contaminant genomic DNA in the RNA-seq library. We checked that our results are robust to this possible artefact (see Additional file 1: Text S5).

### Analysis of intron retention in humans

Braunschweig et al. [29] analyzed 52 RNA-seq samples from different tissues and cell types to quantify intron retention in human genes. For each gene, they selected one representative transcript, based on Ensembl annotations. Their initial dataset includes 202,973 introns from 20,959 protein-coding genes (Additional file 1: Tables S6 and S8 from Braunschweig et al. [29]). We computed the average gene expression level of each gene over the 52 samples, using data provided by the authors. We excluded data from genes that are not mapped on chromosomes of the reference genome assembly (n = 18,546 introns from 2185 genes annotated on unmapped contigs or additional haplotypes) or for which expression data were not available (n = 4844 introns from 871 genes).

To analyze the AS rate according to NMD visibility, we also excluded from their dataset all introns located within UTRs or within truncated CDS (i.e. CDS lacking start or stop codon or containing an internal stop codon): n = 10,780 introns from 912 genes. The final dataset includes 170,015 introns from 16,991 genes.

For each intron, Braunschweig et al. [29] quantified retention rates in all samples where it showed sufficient read depth (>10 reads spanning each flanking exon boundary). Among the 170,015 introns, we excluded those corresponding to minor splice forms (i.e. with an IR rate ≥ 50%, n = 580 introns), and selected all those for which the retention rate had been quantified in at least ten samples. For each of the selected introns (n = 118,703), we computed the average retention rate over all available samples (median = 38 samples).

### Analysis of ASSV in humans

We estimated ASSV frequencies in 25 human tissues and cell lines, using 110 publicly available RNA-seq samples (Additional file 1: Table S3), corresponding to a representative subset of the samples analyzed by Braunschweig et al. [29]. To increase comparability among samples, for paired-end data we analyzed only the first read of the pair and stranded samples were treated as unstranded. We aligned the RNA-seq data on the human genome (hg38 assembly, downloaded from Ensembl release 84) using TopHat 2.0.4 with the following options: minimum intron size for junction discovery = 40 nucleotides (nt), maximum intron size = 1 million nt, maximum one mismatch per read segment, anchor size 8 nt, no mismatches allowed in the anchor region, no coverage search. To aid the spliced read mapping process, we provided as an input for TopHat the set of introns annotated in Ensembl release 84, with the –j option. We re-estimated the splice junction frequencies using uniquely mapping reads, annotated with the NH:i:1 tag in the original TopHat alignments. For each tissue/cell line, we combined read counts from all available samples.

For each intron from Braunschweig dataset (see above), we evaluated whether its 5′ or 3′ splice site were connected with alternative splice sites. We note E1 and E2 the annotated splice sites that border the intron, in 5′-3′ orientation. In a given tissue (*i*), we note *nE1E2*_*i*_ the number of spliced reads corresponding to the annotated splicing event, *nEaE2*_*i*_ the number of spliced reads that connect other 5′ splice sites of the same gene with the 3′ splice site E2, and *nE1Ea*_*i*_ the number of spliced reads that connect the 5′ splice site E1 with other 3′ splice sites of the same gene. We then computed the ASSV frequency:

$$ ASS{V}_i=\left( nE aE{2}_i+ nE 1E{a}_i\right)/\left( nE 1E{2}_i+ nE aE{2}_i+ nE 1E{a}_i\right) $$

For a given intron, this parameter was computed only in tissues with sufficient read depth ((*nE1E2*_*i*_ + *nEaE2*_*i*_ + *nE1Ea*_*i*_) > 10 reads). We excluded 3075 introns corresponding to minor splice forms (i.e. mean ASSV rate ≥ 50%) and selected all introns for which the ASSV rate had been quantified in at least ten tissues. For each of the selected introns (n = 102,697), we computed the average ASSV rate over all available samples (median = 22 samples).

Note that this definition of ASSV includes any splicing event that connects a donor (or acceptor) of the annotated intron, to an alternate acceptor (or respectively donor) in the same gene. This definition encompasses many different types of AS events: not only alternative 3′ or 5′ splice site usage (as shown in Fig. 1c for paramecia), but also exon skipping, alternative initial/terminal exons or mutually exclusive exons [40] (Additional file 1: Figure S12).

### Definition of NMD-invisible alternative splicing events in humans

In mammals, NMD is able to recognize and degrade PTC-containing transcripts only if the PTC occurs more than 50 nucleotides upstream of the last exon-exon junction [6, 49]. Hence, alternative splicing events (IR or ASSV) affecting last introns were classified as NMD-invisible, whereas the other were classified as potentially NMD-visible.

### Analysis of polymorphism at splice sites of human introns

For each of the 170,015 introns located within coding regions, we analyzed patterns of polymorphism in the vicinity of its donor splice site (last 20 bp from the upstream exon and first 30 bp of the intron) and of its acceptor splice site (last 30 bp of the intron and first 20 bp from the downstream exon), using polymorphism data from the 1000 Genomes Project (phase 3; ftp://ftp.1000genomes.ebi.ac.uk/vol1/ftp/release/20130502/) [50]. In total, our dataset includes 447,659 SNPs (0.026 SNP per bp), among which 437,080 (97.6%) with DAF information.

## Additional files

Additional file 1: Includes Text S1–S4, Figures S1–S13, and Tables S1–S3: **Text S1.** Definition of canonical splice forms. **Text S2.** Regulation of splicing factors by AS-NMD in paramecia. **Text S3.** Signatures of selective pressure against splicing errors. **Text S4.** Quantification of the proportion of splicing errors: extended model. **Text S5.** Estimates of IR rate are robust to possible contamination by genomic DNA. **Figure S1.** Impact of NMD on observed IR rates: comparison of biological replicates. **Figure S2.** Impact of NMD on observed PCI splicing rates: comparison of biological replicates. **Figure S3.** Distribution of AS rate in WT cells. **Figure S4.** NMD-sensitive introns in *P. tetraurelia* SRSF-like genes. **Figure S5.** Relationship between AS rate expression level, for NMD-visible or NMD-invisible splicing events. **Figure S6.** Splicing rate of PCIs according to their length. **Figure S7.** Relationship between AS rate and expression level in human genes, for NMD-visible or NMD-invisible AS events. **Figure S8.** Variation in SNP density at splice sites and flanking third codon positions according to gene expression level. **Figure S9.** The fraction of introns with consensus splice signals does not vary with IR rate. **Figure S10.** Signatures of selective pressure against cryptic splicing signals in *P. tetraurelia*. **Figure S11.** Somatic knockouts of UPF1A and UPF1B genes. **Figure S12.** Common forms of AS in humans. **Figure S13.** Read depth in intergenic regions according to the expression level of flanking genes. **Table S1.** Summary of RNA-seq samples. **Table S2.** Number of introns or cryptic introns showing evidence of AS in RNA-seq samples from WT or NMD-deficient paramecia. **Table S3.** RNA-seq libraries analyzed to quantify ASSV in human. (PDF 1759 kb)
