# Supplementary material for: The fitness cost of mis-splicing is the main determinant of alternative splicing patterns
Source: Genome Biol. 2017 Oct 30;18:208. doi: 10.1186/s13059-017-1344-6 (PMC5663052; doi:10.1186/s13059-017-1344-6)
Supplement: Supplementary file 1 — Includes Text S1–S4, Figures S1–S13, and Tables S1–S3: Text S1. Definition of canonical splice forms. Text S2. Regulation of splicing factors by AS-NMD in paramecia. Text S3. Signatures of selective pressure against splicing errors. Text S4. Quantification of the proportion of splicing errors: extended model. Text S5. Estimates of IR rate are robust to possible contamination by genomic DNA. Figure S1. Impact of NMD on observed IR rates: comparison of biological replicates. Figure S2. Impact of NMD on observed PCI splicing rates: comparison of biological replicates. Figure S3. Distribution of AS rate in WT cells. Figure S4. NMD-sensitive introns in P. tetraurelia SRSF-like genes. Figure S5. Relationship between AS rate expression level, for NMD-visible or NMD-invisible splicing events. Figure S6. Splicing rate of PCIs according to their length. Figure S7. Relationship between AS rate and expression level in human genes, for NMD-visible or NMD-invisible AS events. Figure S8. Variation in SNP density at splice sites and flanking third codon positions according to gene expression level. Figure S9. The fraction of introns with consensus splice signals does not vary with IR rate. Figure S10. Signatures of selective pressure against cryptic splicing signals in P. tetraurelia. Figure S11. Somatic knockouts of UPF1A and UPF1B genes. Figure S12. Common forms of AS in humans. Figure S13. Read depth in intergenic regions according to the expression level of flanking genes. Table S1. Summary of RNA-seq samples. Table S2. Number of introns or cryptic introns showing evidence of AS in RNA-seq samples from WT or NMD-deficient paramecia. Table S3. RNA-seq libraries analyzed to quantify ASSV in human. (PDF 1759 kb) [file 13059_2017_1344_MOESM1_ESM.pdf]

Supplementary Material

The fitness cost of mis-splicing is the main determinant of alternative splicing patterns

Baptiste Saudemont, Alexandra Popa, Joanna L. Parmley, Vincent Rocher, Corinne Blugeon, Anamaria Necsulea, Eric Meyer, Laurent Duret

Supplemental Text S1: Definition of canonical splice forms 2

Supplemental Text S2: Regulation of splicing factors by AS-NMD in paramecia 2

Identification of SRSF-like genes 3

Supplemental Text S3: Signatures of selective pressure against splicing errors 3

Supplemental Text S4: Quantification of the proportion of splicing errors: extended model 3

Supplemental Text S5: Estimates of IR rate are robust to possible contamination by genomic DNA 5

References 7

Supplemental Figures 8

Supplemental Figure S1: Impact of NMD on observed IR rates: comparison of biological replicates. 8

Supplemental Figure S2: Impact of NMD on observed PCI splicing rates: comparison of biological replicates. 9

Supplemental Figure S3: Distribution of AS rate in WT cells. 10

Supplemental Figure S4: NMD-sensitive introns in P. tetraurelia SRSF-like genes. 11

Supplemental Figure S5: Relationship between AS rate expression level, for NMD-visible or NMD-invisible splicing events. 12

Supplemental Figure S6: Splicing rate of PCIs according to their length. 13

Supplemental Figure S7: Relationship between AS rate and expression level in human genes, for NMD-visible or NMD-invisible AS events. 14

Supplemental Figure S8: Variation in SNP density at splice sites and flanking third codon positions according to gene expression level. 15

Supplemental Figure S9: The fraction of introns with consensus splice signals does not vary with IR rate 16

Supplemental Figure S10: Signatures of selective pressure against cryptic splicing signals in *P. tetraurelia*. 17

Supplemental Figure S11: Somatic knockouts of UPF1A and UPF1B genes 18

Supplemental Figure S12: Common forms of alternative splicing in humans 19

Supplemental Figure S13: Read depth in intergenic regions according to the expression level of flanking genes. 19

Supplemental Tables 20

Supplemental Table S1: Summary of RNAseq samples. 20

Supplemental Table S2: Number of introns or cryptic introns showing evidence of alternative splicing in RNAseq samples from WT or NMD-deficient paramecia. 21

Supplemental Table S3: RNAseq libraries analyzed to quantify ASSV in human. 22

Supplemental Text S1: Definition of canonical splice forms

The classification of splice variants relies on the definition of a canonical form (Fig1C): the distinction between a "cryptic intron" and a "retained intron" depends on which variant is considered as the reference. Here we decided to define the canonical form as the one that is the most abundant in WT cells. The underlying assumption is that for most genes, there exists a dominant transcript, and that the other variants (functional or not) are quantitatively minor. To test whether this assumption is correct, we measured the rate of splice variation of individual introns in WT cells. For a given intron, the rate of splice variation is defined as the proportion of reads that differ from the annotated spliced form (either IR or ASSV), among all reads spanning both flanking exons (Fig1C). To avoid artefacts owing to gene annotation errors, we restricted this analysis to introns for which the spliced form is observed in at least one read of the WT samples. And to limit the variance in the measurement, we analyzed introns overlapped by at least 50 sequence reads. This subset represents 44% of all annotated introns, and hence is expected to be representative of the entire genome. In 99.4% of cases, the annotated intron corresponds to the major splice form (Supplemental Figure S3). On average, the rate of splicing variations is 3% (median 1%), and for 97.8% of introns, minor variants correspond to less than 20% of all spanning reads. Thus, in the huge majority of cases, there exists one major splice form that strongly predominates over other variants. Hence, we considered the major splice form (in WT samples) to be the canonical one.

Supplemental Text S2: Regulation of splicing factors by AS-NMD in paramecia

Our results indicate that the pattern of alternative splicing in paramecium is dominated by splicing errors. But of course, this does not exclude that a small fraction of genes might be subject to functional alternative splicing events. In particular, there is evidence from different model organisms that AS-NMD can play an important role in the regulation of genes encoding splicing factors (Lareau et al., 2007; Ni et al., 2007). To test whether this is also the case in paramecia, we searched for homologs of arginine-serine rich splicing factor 5 (SRSF5), which has been shown to be regulated by AS-NMD both in fungi and animals (Lareau and Brenner, 2015). We identified 11 SRSF5-homologs, which can be classified into 4 distinct clades (which we refer to as A, B, C and D). As in other eukaryotes, these genes are expressed at high levels (Supplemental Figure S4). They contain two to four introns (the intron/exon structure is conserved within clades, but differ between clades). All these genes include at least one intron showing extremely weak intrinsic splicing efficiency, with rates of IR or ASSV ranging from 23% to 76% (mean = 48%) in NMD-deficient cells. In most cases, there is only one such intron (the only exception is gene C3, which has two), and it is conserved among paralogs from a same clade (Supplemental Figure S4). The abundance of these splice variants is strongly sensitive to NMD (Supplemental Figure S4). These observations indicate that in paramecia, as in many other eukaryotes, genes encoding splicing factors contain introns with very weak intrinsic splicing efficiency, which can trigger the regulation of gene expression by AS-NMD. It should be stressed that the levels splicing variation observed in the 12 weak introns from SRSF5-homologs is extremely high: compared to introns from genes with similar expression levels, 11 of these introns are in the top 1% with highest IR or ASSV rate and the last one is in the top 5% (Supplemental Figure S4). This

suggests that a very high IR or ASSV rate is probably a good signature to predict genes subject to functional alternative splicing.

Identification of SRSF-like genes

Arginine-serine rich splicing factor 5 (SRSF5) and its two closely related paralogs (SRSF4 and SRSF6) have been shown to be regulated by AS-NMD both in fungi and animals (Lareau and Brenner, 2015). We compared human SRSF4, SRSF5 and SRSF6 proteins (Uniprot accession numbers Q08170, Q13243 and Q13247) against all paramecium proteins using BLASTP, with an E-value threshold of 10^{-3} (Altschul et al., 1997). We identified 11 hits common to the three paralogs (Gene accession numbers: GSPATG00039656001, GSPATG00037425001, GSPATG00002543001, GSPATG00001514001, GSPATG00019872001, GSPATG00009396001, GSPATG00025558001, GSPATG00030796001, GSPATG00003006001, GSPATG00001197001, GSPATG00005005001). We manually corrected several errors in the original gene annotations, using RNAseq data from WT samples to identify the correct exon junctions. Note that these annotation errors did not affect the 12 introns that we identified as being subject to AS-NMD (Supplemental Figure S4), except intron 1 from GSPATG00030796001. The phylogenetic tree was inferred from the protein multiple with PHYML (Guindon and Gascuel, 2003), using the SEAVIEW software (Gouy et al., 2010).

Supplemental Text S3: Signatures of selective pressure against splicing errors

We detected cryptic intron splicing in more than 30% of paramecium genes. Their size distribution is very similar to that of introns, with 97.1% of them between 20 and 35 bp (mean= 26.0 bp) (Fig1B). We have previously shown that in paramecium, as in many other eukaryotes, introns are under selective pressure to ensure that NMD can detect and degrade transcripts in case of intron retention (Jaillon et al. 2008). This constraint leads to a deficit in introns of length multiple of three (3n introns), lacking in-frame stop codons (Jaillon et al. 2008). By definition cryptic introns are located within coding regions, and hence do not contain any in-frame stop codon. Interestingly, we observe a very strong deficit of 3n cryptic introns (Fig1B; overall, only 18% of cryptic introns are of length multiple of 3), which suggests that cryptic introns that preserve the reading frame are counter-selected. This is consistent with the hypothesis that cryptic introns generally result from errors of the splicing machinery, and that such errors are more costly in the case of 3n cryptic introns because they are not detectable by NMD.

In paramecia, splice signals are characterized by a strong conservation of the first and last 3 bp of introns (71% of introns match the consensus sequence: [GTA$(N)_n$TAG]). The analysis of the distribution of distances between GTA and TAG triplets within coding exons shows a deficit of TAG downstream of GTA, specifically in a window 20 to 35 bp, which corresponds to the length of *bona fide* introns (Supplemental Figure S10). This indicates that strong cryptic splice sites are counter-selected within exons, in agreement with the hypothesis that they are generally deleterious.

Supplemental Text S4: Quantification of the proportion of splicing errors: extended model

Our estimates of the proportion of AS events corresponding to splicing errors are based on the assumption that the rate of functional AS events (AS^f) does not vary with gene expression (see Equation (3) in the main text for more details). To test whether this assumption could bias our results, we explored a more complex model, where we considered that AS^f might vary with expression level. Let us note *k,* the ratio of the functional AS rate in a given bin of expression (i) over the AS rate in highly constrained genes:

$$k = \frac{AS_i}{AS_h} = \frac{AS_i^f}{AS_h^f}$$

Thus, the proportion of splicing errors in expression bin (i) given by Equation (4) becomes:

$$P_i^e = 1 - \frac{k}{r_i} \qquad (5)$$

Equation (5) shows that if the rate of functional AS was negatively correlated with the gene expression level (i.e. if k>1), then Equation (4) would lead to overestimate the proportion of splicing errors (and conversely if k<1).

Estimates of the proportion of variants resulting from splicing errors (for a gene with median expression level) are given below for different values of k:

	r_i	k=1	k=2	k=4	k=8
IR	12.0	92%	83%	67%	33%
ASSV	20.3	95%	90%	80%	61%
PCI	49.3	98%	96%	92%	84%

The main conclusion reported in the main text (based on the assumption that k=1) is that in a median gene, the vast majority of AS events correspond to errors. Thus, this conclusion would remain valid up to k=4 for IR and k=8 for ASSV or PCI. We will discuss below whether such values of k are plausible or not.

First, let us precise one point of terminology. Two types of functional AS events can be distinguished:

- AS events that lead to the production of functional protein variants.
- AS events that do not produce functional proteins, but that contribute to the regulation of gene expression level via AS-NMD.

We will hereafter refer to the first type as "AS-FPV" (functional protein variants) and the second one as AS-NMD (regulatory function). The rate of functional AS can therefore be decomposed as:

$$AS^f = AS^{FPV} + AS^{NMD}$$

Thus, if k>1, this implies that gene expression level is negatively correlated either with AS^{FPV} or with AS^{NMD}. As mentioned in the main text, our analyses rule out the latter hypothesis. Indeed, we observed a strong negative relationship between AS rate and gene expression level, both for NMD-visible and NMD-invisible splicing variants (which, by definition, cannot contribute to AS-NMD). This pattern is observed both in paramecium (Fig. 4) and in human (Suppl. Fig. S7). Hence, if k > 1, this must be due to AS-FPV and not to AS-NMD.

It is in principle possible that AS^{FPV} vary with expression level. However, AS^{FPV} would have to be quite high to affect significantly the estimates of splicing error rates reported in the main text. For instance, as shown in the above table, a value k=4 implies that in a median gene, 20% of ASSV variants are functional (compared to 5% if k=1). Thus, if k=4, this would imply that at least 15% (and possibly up to 20%) of ASSV variants correspond to AS-FPV events. For IR variants, this proportion would have to be even higher (25% to 33%).

This is in contradiction with numerous lines of evidence indicating that AS-FPV represents only a tiny fraction of all AS events (reviewed in Tress et al. 2017a,b). Proteomic studies (covering > 100 distinct tissues and cell lines) showed that at the protein level, 98% of genes produce one single dominant isoform: only 0.6% of all annotated AS events lead to the production of a detectable amount of protein (Abascal et al. 2015). Of course, this does not exclude the possibility that many genes could produce functional protein variants at low levels, below the limit of detection of proteomic analyses. However, if such minor isoforms were functional, one would expect them to display the same signatures of protein functionality as the protein variants that have been detected in proteomic studies. Yet, the bulk of AS variants identified in transcriptomic studies show clearly distinct features compared to the ones that have been validated at the protein level:

- 70% of them disrupt the domain structure of proteins (compared to 15% for validated variants) (Abascal et al. 2015)
- 58% of them shift the reading frame (compared to 66% expected by chance) (Pickrell et al. 2010) whereas this is the case for only 4% of validated variants (Tress et al. 2017b)
- comparative transcriptomic analyses revealed that only 1%-3% of exon-skipping events detected with RNA-seq are conserved beyond mammals (Merkin et al., 2012; Barbosa-Morais et al., 2012), whereas the analysis of 60 exon-skipping events validated by proteomics data revealed that 100% of them are conserved from mammals to bony fish (Abascal et al. 2015).

All these observations indicate that AS-FPV represents at most a few percent of all AS events, which implies that k must be lower than 4.

Our observations provide additional evidence indicating that AS-FPV cannot account for the relationship between AS rate and gene expression level. Indeed, the fact that 96% of the variants validated by proteomics data preserve the reading frame (Tress et al. 2017b) demonstrates that AS-FPV is extremely rare among AS events that induce PTCs. Thus, if AS-FPV contributed substantially to the increase in AS rate in weakly expressed genes (as expected if k=4), then one would expect this increase to be stronger among frame-preserving AS events than among PTC-inducing events. As shown in Figure 4, this is clearly not the case: the increase in AS rate with decreasing expression level is in fact stronger for PTC-inducing events than for the frame-preserving ones (NMD-invisible).

Thus, our main conclusion remains robust for plausible values of k.

Supplemental Text S5: Estimates of IR rate are robust to possible contamination by genomic DNA

It is in principle possible that a small fraction of RNAseq sequence reads correspond to contaminant genomic DNA fragments. Contaminant genomic reads spanning an intron are counted as unspliced reads, and therefore lead to bias the measure of IR rate. This artefact can potentially contribute to the observed relationship between IR rate and expression level: in highly expressed genes, the fraction of reads corresponding to contaminant DNA is expected to be negligible. But in weakly expressed genes, contaminant DNA reads might significantly inflate the observed IR rate.

To quantify the potential amount of DNA contamination in our RNAseq libraries, we analyzed read depth in intergenic regions. Given that UTRs and non-coding RNA genes are poorly annotated in the genome of paramecium, we defined here intergenic regions as the interval between coding regions of consecutive protein-coding genes. We measured the read depth at the center of each of these intervals. The genome of Paramecium is very compact (70% coding), with very short intergenic regions (mean = 337.1 bp, median = 156 bp). Thus, UTRs may represent a substantial fraction of the length of intergenic regions. Moreover, transcriptional read-through may lead to the production of *bona fide* RNA reads beyond the canonical polyadenylation site. Hence, it is expected that the RNAseq read depth in intergenic regions should depend on the expression level of their flanking genes. And indeed, we observed that the read depth in intergenic regions is strongly correlated to the level of expression of flanking genes (Supplemental Figure S13).

In the subset of intergenic regions flanked by genes with very low expression, the average read depth at their center is 0.58 in NMD-deficient samples (Supplemental Figure S13) and 0.59 in WT samples (not shown). The level of DNA contamination is not expected to depend on gene expression level. Hence, the read depth that is potentially due to DNA contamination is at most 0.59. It should be noted that this corresponds to an upper estimate, because some intergenic regions may contain unannotated genes (protein-coding genes or non-coding RNA genes).

We re-estimated IR rates in NMD-visible introns for each expression bin, after subtracting the number of unspliced reads potentially corresponding to DNA contaminants (0.59 reads per intron). This lower boundary of the true IR rate is indicated by dashed lines in Supplemental Figure S5A. This figure shows that even after controlling for potential DNA contaminants, there remains a strong negative relationship between IR rates and expression level. Hence, DNA contamination cannot be the cause of the correlation between IR rates and expression level.

It should also be noted that there is a strong negative relationship between AS rate and expression level, not only for IR, but also for ASSV and cryptic intron splicing (Figure 3). Obviously, these two latter categories cannot be explained by DNA contamination since, by definition, they correspond to sequence reads with evidence of splicing. Hence, our conclusion that AS rates correlate negatively with expression level is robust to possible issues of DNA contamination.

References

Abascal F, Ezkurdia I, Rodriguez-Rivas J, Rodriguez JM, del Pozo A, Vázquez J, et al. 2015. Alternatively Spliced Homologous Exons Have Ancient Origins and Are Highly Expressed at the Protein Level. *PLOS Comput. Biol.* **11**:e1004325.

Altschul, S.F., Madden, T.L., Schäffer, A.A., Zhang, J., Zhang, Z., et al. 1997. Gapped BLAST and PSI-BLAST:a new generation of protein database search programs. *Nucleic Acids Res* **25**: 3389–3402.

Barbosa-Morais NL, Irimia M, Pan Q, Xiong HY, Gueroussov S, Lee LJ, et al. 2012. The evolutionary landscape of alternative splicing in vertebrate species. *Science* **338**:1587–93.

Gouy, M., Guindon, S., and Gascuel, O. 2010. SeaView version 4: A multiplatform graphical user interface for sequence alignment and phylogenetic tree building. *Mol. Biol. Evol.* **27**: 221–224.

Guindon, S. and Gascuel, O. 2003. A simple, fast, and accurate algorithm to estimate large phylogenies by maximum likelihood. *Syst. Biol.* **52**: 696–704.

Lareau, L.F. and Brenner, S.E. 2015. Regulation of Splicing Factors by Alternative Splicing and NMD Is Conserved between Kingdoms Yet Evolutionarily Flexible. *Mol. Biol. Evol.* **32**: 1072–1079.

Lareau, L.F., Inada, M., Green, R.E., Wengrod, J.C., and Brenner, S.E. 2007. Unproductive splicing of SR genes associated with highly conserved and ultraconserved DNA elements. *Nature* **446**: 926–9.

Merkin J, Russell C, Chen P, Burge CB. 2012. Evolutionary dynamics of gene and isoform regulation in Mammalian tissues. *Science* **338**:1593–9.

Ni, J.Z., Grate, L., Donohue, J.P., Preston, C., Nobida, N., et al. 2007. Ultraconserved elements are associated with homeostatic control of splicing regulators by alternative splicing and nonsense-mediated decay. *Genes Dev.* **21**: 708–18.

Pickrell JK, Pai A a, Gilad Y, Pritchard JK. 2010. Noisy splicing drives mRNA isoform diversity in human cells. *PLoS Genet.* **6:**e1001236.

Tress ML, Abascal F, Valencia A. 2017a. Most Alternative Isoforms Are Not Functionally Important. *Trends Biochem. Sci.* **42**:408–10

Tress ML, Abascal F, Valencia A. 2017b. Alternative Splicing May Not Be the Key to Proteome Complexity. *Trends Biochem. Sci.* **42**:98–110.

Supplemental Figures

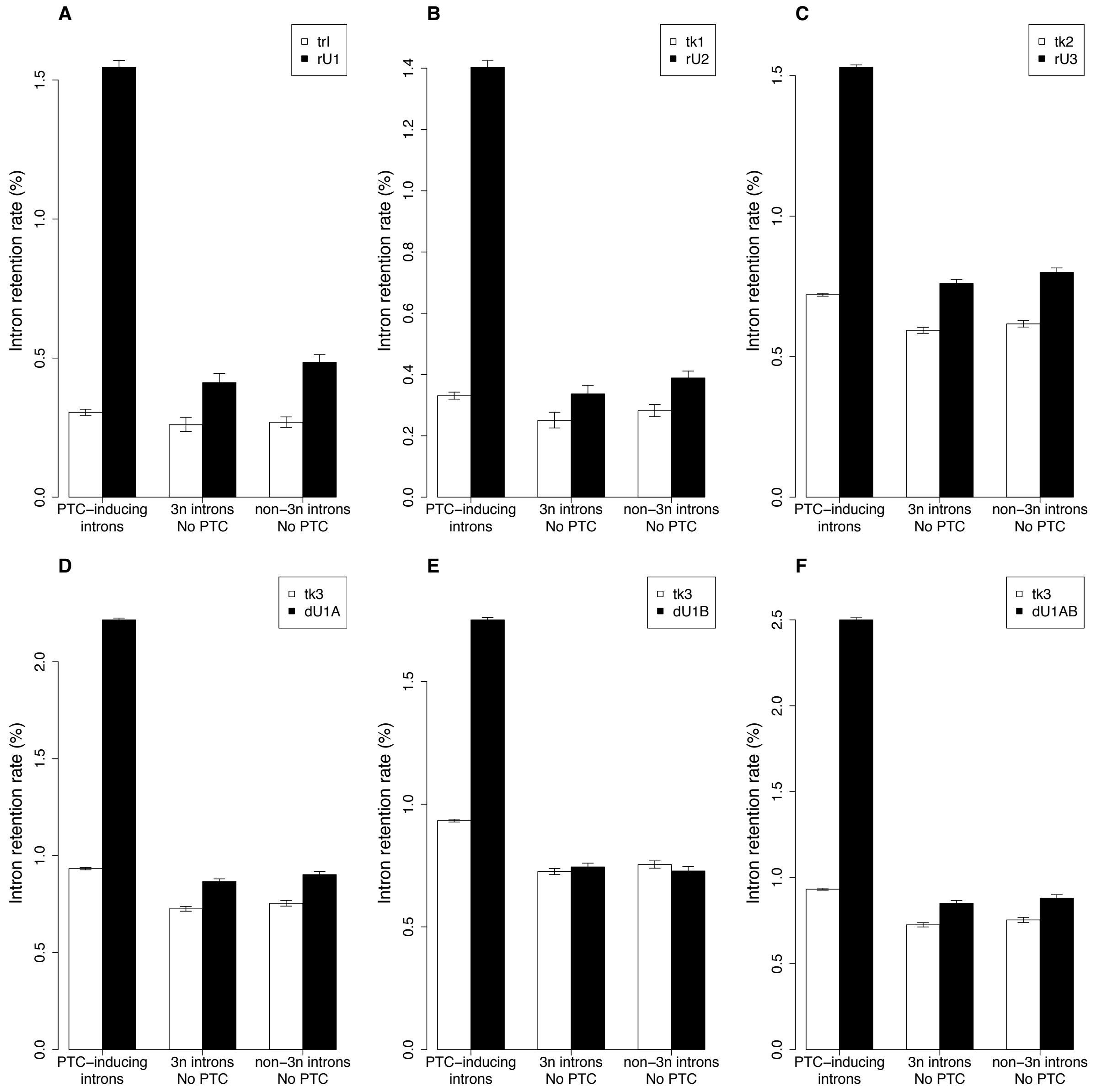

Supplemental Figure S1: Impact of NMD on observed IR rates: comparison of biological replicates.

N= 65,159 introns. IR events are classified into three groups according to their NMD-visibility: PTC-inducing events (i.e. NMD-visible); events that do not introduce frameshift or PTC (3n no PTC); events that create a frameshift but without introducing a PTC (non-3n no PTC). IR rates in WT and in NMD-deficient cells were computed globally within each bin, as the proportion of IR reads among all reads spanning introns from that bin. Error bars represent the 95% confidence interval of this proportion. Results of individual biological replicates are displayed in the different panels (A-F). NMD-silencing experiments (black bars): rU1: RNAi against UPF1A+UPF1B, rU2: RNAi against UPF2, rU3: RNAi against UPF3, dU1A: somatic deletion of UPF1A, dU1B: somatic deletion of UPF1B, dU1AB: somatic deletion of UPF1A + UPF1B. Control experiments (white bars): tk1, tk2, tk3: three biological replicates of normal cells fed with K. pneumoniae; trI: RNAi against ICL7a (a gene not involved in NMD).

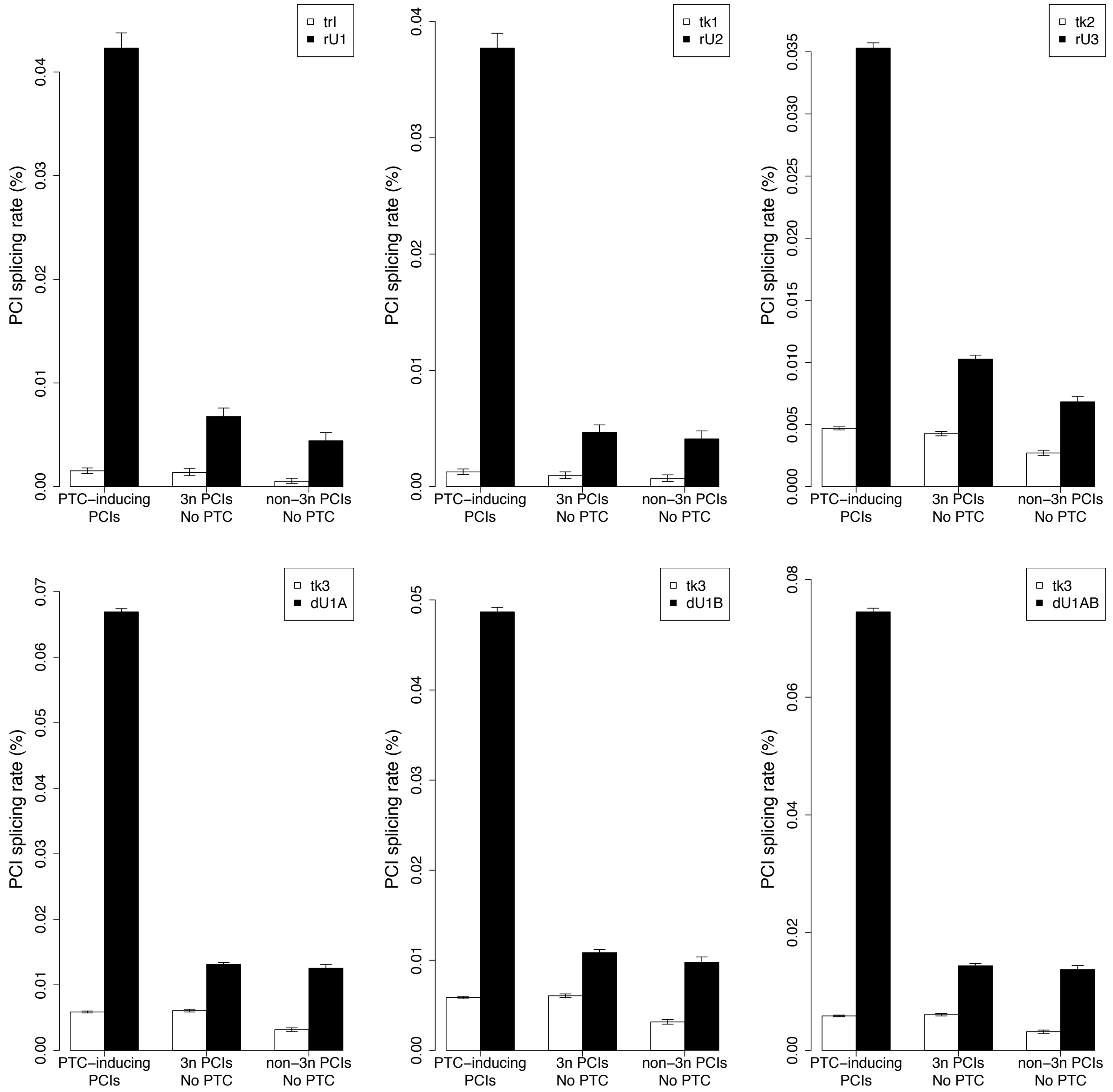

Supplemental Figure S2: Impact of NMD on observed PCI splicing rates: comparison of biological replicates.

N= 1,383,067 PCIs. Cryptic intron splicing events are classified into three groups according to their NMD-visibility: PTC-inducing events (i.e. NMD-visible); events that do not introduce frameshift or PTC (3n no PTC); events that create a frameshift but without introducing a PTC (non-3n no PTC). PCI splicing rates in WT and in NMD-deficient cells were computed globally within each bin, as the proportion of spliced reads among all reads spanning PCIs from that bin. Error bars represent the 95% confidence interval of this proportion. Results of individual biological replicates are displayed in the different panels (A-F). NMD-silencing experiments (black bars): rU1: RNAi against UPF1A+UPF1B, rU2: RNAi against UPF2, rU3: RNAi against UPF3, dU1A: somatic deletion of UPF1A, dU1B: somatic deletion of UPF1B, dU1AB: somatic deletion of UPF1A + UPF1B. Control experiments (white bars): tk1, tk2, tk3: three biological replicates of normal cells fed with K. pneumoniae; trI: RNAi against ICL7a (a gene not involved in NMD).

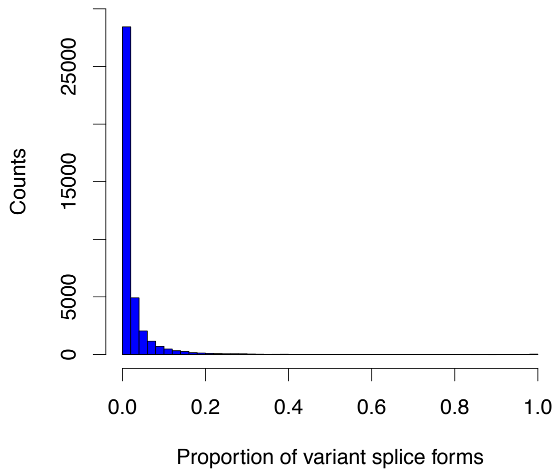

Supplemental Figure S3: Distribution of AS rate in WT cells.

N=39,461 annotated introns, covered by at least 50 sequence reads in WT cells, and for which at least one read corresponds to the annotated splicing form. The AS rate at a given intron is defined as the ratio $(n_2+n_3)/(n_1+n_2+n_3)$*, where* n_1 *is the number of reads matching with the annotated splicing event,* n_2 *is the number of reads showing splicing with alternative splice sites, and* n_3 *the number of reads showing intron retention, and* $(n_1+n_2+n_3)$ *is the total number of reads spanning both flanking exons (see Fig1C).*

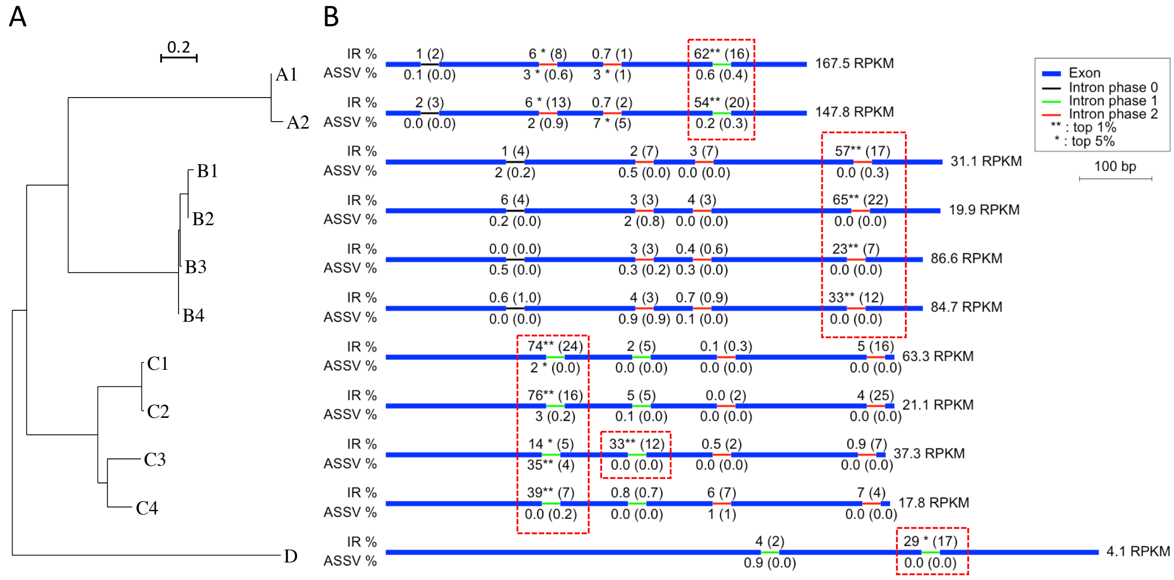

Supplemental Figure S4: NMD-sensitive introns in P. tetraurelia SRSF-like genes.

(A) Phylogeny of SRSF-like genes identified in P. tetraurelia, based on protein sequence alignment (computed with Phyml, using LG model). These 11 genes can be classified into 4 clades (named here A, B, C and D). (B) The exon/intron structure is conserved within each clade (introns are located at conserved position, in the same phase), but differs between clades. IR and ASSV rates measured in NMD-deficient cells are indicated respectively above and below each intron. Values that are above the 5% or 1% highest rates observed among introns from genes with similar expression level are indicated by '' or '**' respectively. IR and ASSV rates measured in WT cells are indicated in parenthesis. Expression level in WT cells (RPKM) is indicated. All these genes contain introns with levels of IR or ASSV that are extremely high and sensitive to NMD inactivation (dashed red boxes). In most cases, there is only one such intron, and this intron is shared among paralogs from a same clade. Gene accession numbers: A1: GSPATG00039656001, A2: GSPATG00037425001, B1: GSPATG00002543001, B2: GSPATG00001514001, B3: GSPATG00019872001, B4: GSPATG00009396001, C1: GSPATG00025558001, C2: GSPATG00030796001, C3: GSPATG00003006001, C4: GSPATG00001197001, D: GSPATG00005005001.*

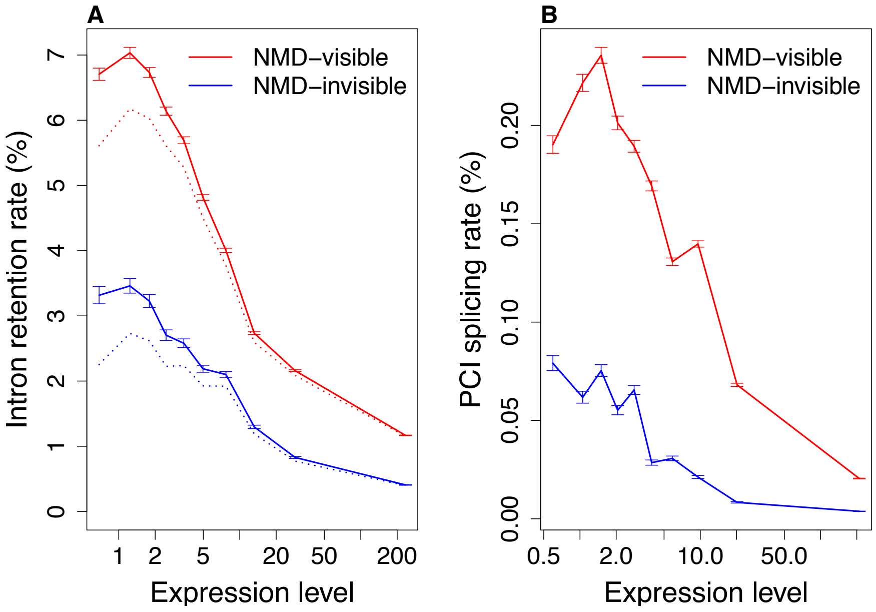

Supplemental Figure S5: Relationship between AS rate expression level, for NMD-visible or NMD-invisible splicing events.

Same as Figure 4, but AS rates measured in NMD-deficient cells. (A) Introns were first classified into two groups according to their NMD-visibility in case of retention events (N= 52,163 NMD-visible introns, in red, and N=12,996 NMD-invisible introns, in blue), and then further grouped into ten bins of equal sample size, according to gene expression levels in WT cells. IR rates (in NMD-deficient cells) were measured globally in each bin. Error bars represent the 95% confidence interval of the proportion of AS reads. Dashed lines correspond to the lower boundary of the IR rate, after subtracting the number of unspliced reads potentially corresponding to DNA contaminants (see Supplemental Text S5). It should be stressed that this dashed line does not represent the true IR rate, but only the upper limit of the potential bias introduced by DNA contamination on estimated IR rates. (B) Same as (A), but for the splicing of PCIs: N= 882,579 NMD-visible PCIs, and N=500,488 NMD-invisible PCIs. Expression levels (RPKM) are represented in log scale.

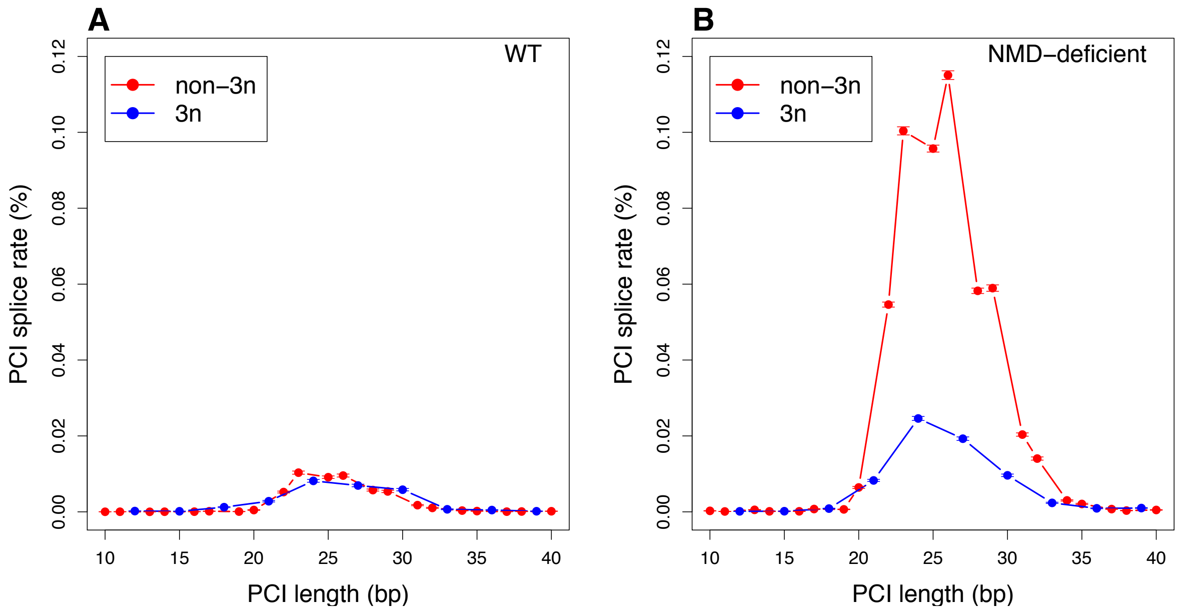

Supplemental Figure S6: Splicing rate of PCIs according to their length.

The definition of PCIs (see main text) was extended here to include all exonic [GT..AG] segments of length 10 to 40 nt (N=2,784,653 PCIs). The splicing rate was computed globally within each size bin, as the proportion of spliced reads among all reads spanning PCIs from that bin. Error bars represent the 95% confidence interval of this proportion (NB: in many cases, error bars are too small to be visible). (A) PCIs splicing rates measured in normal cells. (B) PCIs splicing rates measured in NMD-deficient cells. Frameshifting (non-3n) and non-frameshifting (3n) PCIs are displayed respectively in red and in blue.

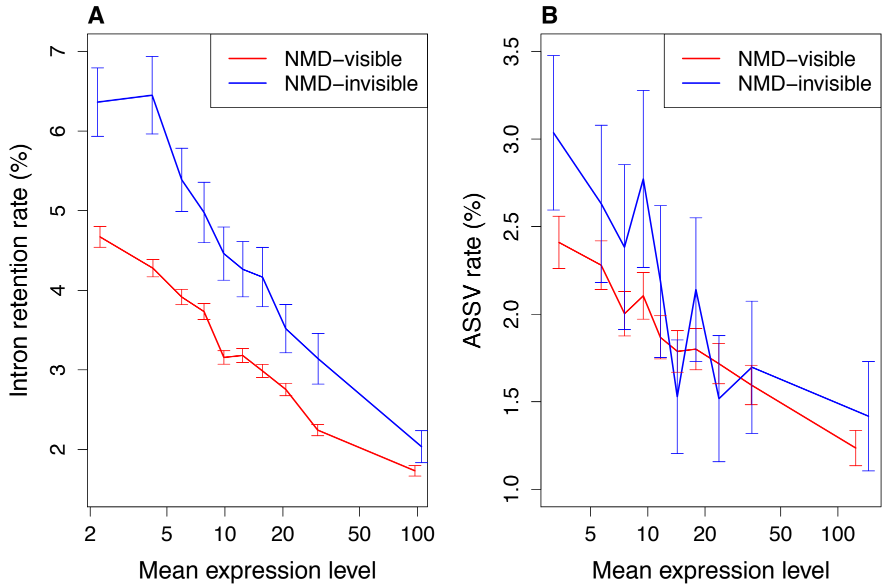

Supplemental Figure S7: Relationship between AS rate and expression level in human genes, for NMD-visible or NMD-invisible AS events.

(A) IR rate (N=118,703 introns). (B) ASSV rate (N=102,697 introns). In each panel, introns were first classified into two groups according to the NMD-visibility of AS events (NMD-visible events, in red, and NMD-invisible events, in blue), and then further grouped into ten bins of equal sample size, according to gene expression levels. We computed the average AS rate (IR or ASSV) over all introns within each bin. Error bars represent the 95% confidence interval of the mean. (B)

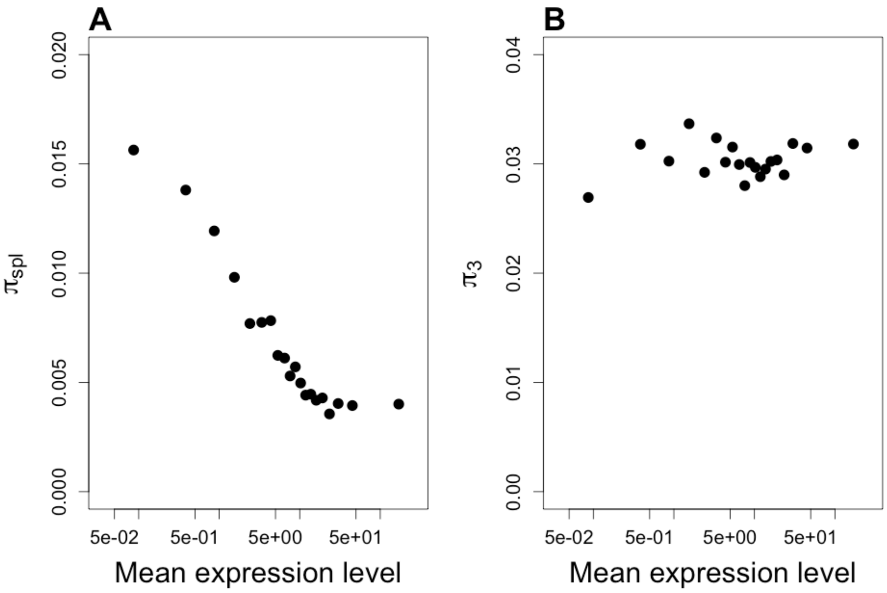

Supplemental Figure S8: Variation in SNP density at splice sites and flanking third codon positions according to gene expression level.

Human introns located between coding exons (N=170,015) were classified into bins of equal sample size according to gene expression levels. SNP density was measured over all introns within each bin. (A) SNP density at splice sites (π_{spl}) is negatively correlated with expression level (R^2=0.89, $p<10^{-9}$). (B) : SNP density at flanking third codon positions (π3; measured over 20 bp within each flanking exons) does not correlate with expression level (R^2=0.04, p=0.38) .

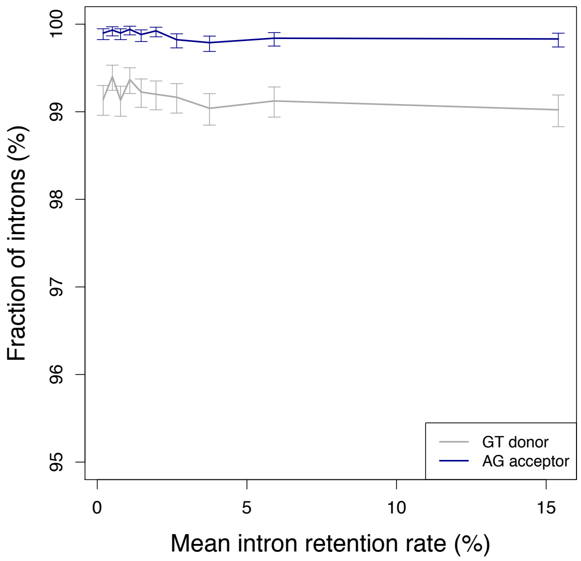

Supplemental Figure S9: The fraction of introns with consensus splice signals does not vary with IR rate.

Human introns were classified into bins of equal sample size according to their average retention rate, and the proportion of introns matching the consensus splice donor (GT), and the proportion of introns matching the consensus splice acceptor (AG) was computed for each bin. Error bars represent the 95% confidence interval of this proportion.

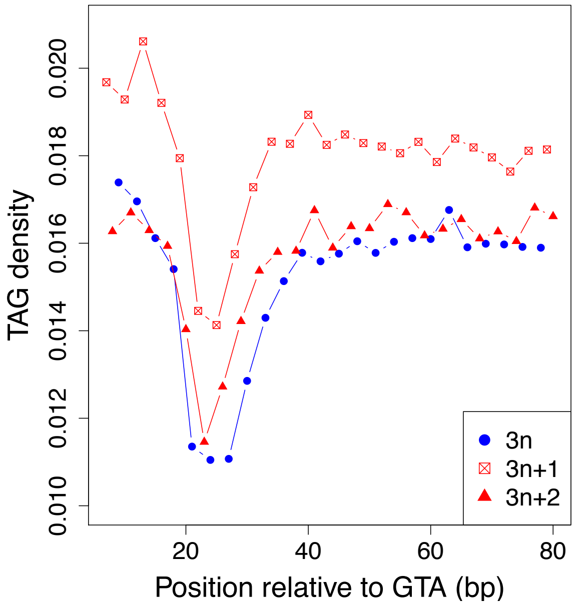

Supplemental Figure S10: Signatures of selective pressure against cryptic splicing signals in *P. tetraurelia*.

Density in TAG trinucleotides within coding regions, according to the distance to upstream GTA trinucleotides located in the same exon. [GTA..TAG] segments of length 3n, or non-3n (3n+1 and 3n+2) are displayed respectively in blue and red.

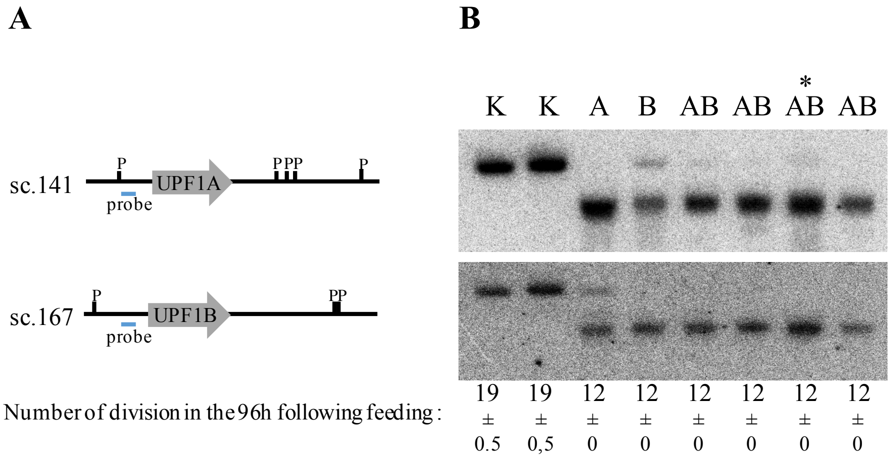

Supplemental Figure S11: Somatic knockouts of UPF1A and UPF1B genes.

(A) *Schematic representation of the UPF1A and UPF1B genes with surrounding PacI (P) sites (scaffolds 141 and 167). The ~470-bp probes used for Southern blots are located ~700 bp upstream of start codons.* ***(B)*** *Southern blot of PacI-restricted genomic DNA samples. The blot was hybridized successively with UPF1A and UPF1B probes. The sizes of PacI fragments from wild-type controls (K, clones fed continuously with Klebsiella bacteria) are 5,582 bp for UPF1A and 9,422 bp for UPF1B. Deletions can be observed by the shift of the band in DNA samples from clones subjected to post-conjugation feeding (A: UPF1A dsRNA; B: UPF1B dsRNA; AB: UPF1A+UPF1B dsRNAs - see Methods). Deletions were mapped by PCR using primers outside the genes, revealing deletion sizes of ~3,800 bp for UPF1A and ~3,250 bp for UPF1B. The double knockout used for RNA-seq is indicated by a star above the lane. The number of divisions of each ex-conjugant clone in the first 96 hrs after conjugation is indicated below each lane.*

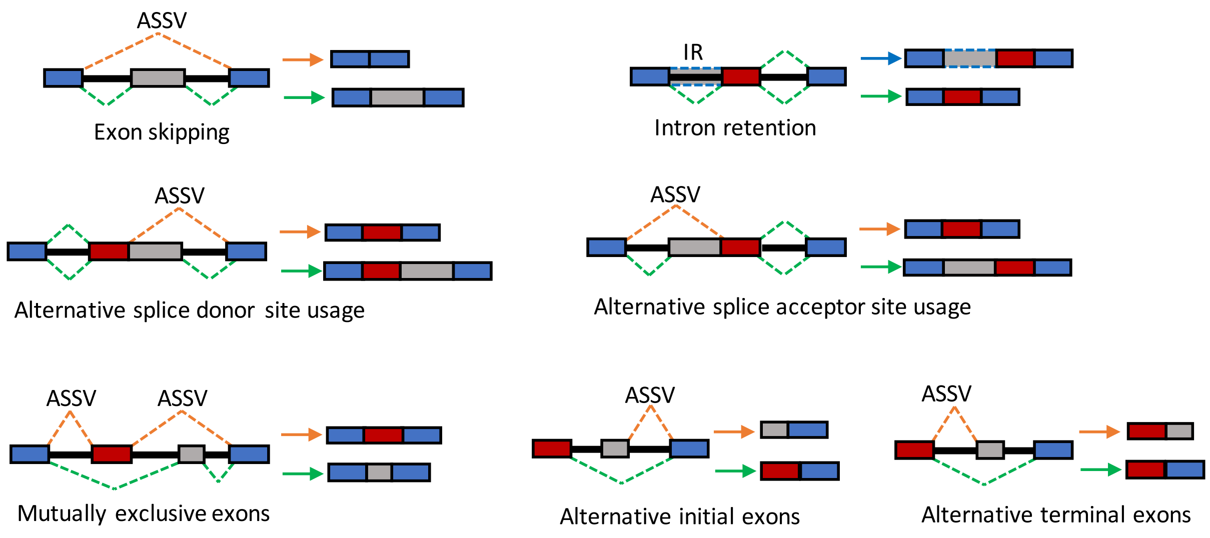

Supplemental Figure S12: Common forms of alternative splicing in humans

The major splice form is indicated in green. ASSV events are indicated in orange. (redrawn from Wang & Burge 2008 RNA 14:802–13).

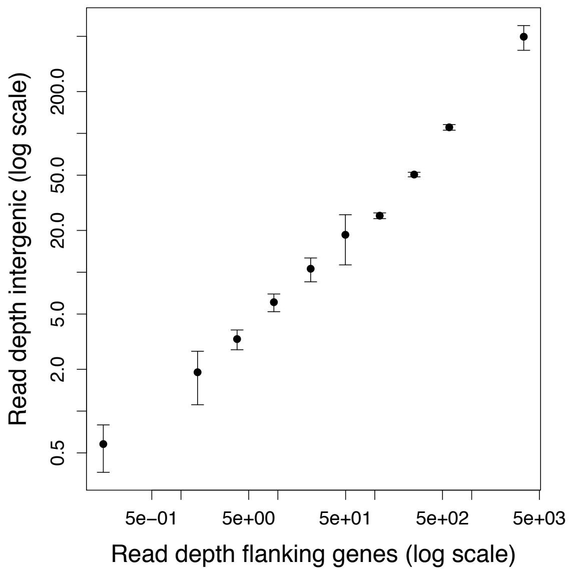

Supplemental Figure S13: Read depth in intergenic regions according to the expression level of flanking genes.

Paramecium intergenic regions (N=38,966) were classified into 10 bins according to the RNAseq read depth in their flanking genes. For each bin, we computed the average read depth at the center of intergenic regions. Error bars correspond to the 95% confidence interval of the mean. This figure shows the results for RNAseq data from NMD-deficient paramecia. We observed the same pattern in WT cells (not shown).

Supplemental Tables

Supplemental Table S1: Summary of RNAseq samples.

Sample ID	Description	Strain	Short ID	Sequencing strategy	Read length	FASTQ file ID	Number of fragments	Raw sequence length (bp)
K	Control: no RNAi (cells fed with Klebsiella)	51	tK1	single-end<br>single-end	75<br>75	AGC_DOSS_7_42DPEAAXX<br>AGC_DOSS_8_42DPEAAXX	9383971<br>8875969	703797825<br>665697675
I	Control: RNAi against ICL7a (a gene not involved in NMD)	51	trI	single-end<br>single-end	75<br>75	AGC_COSS_5_42DPEAAXX<br>AGC_COSS_6_42DPEAAXX	9218957<br>9154609	691421775<br>686595675
A	NMD-deficient: RNAi against UPF1A and UPF1B	51	rU1	single-end<br>single-end	75<br>75	AGC_AOSS_1_42DPEAAXX<br>AGC_AOSS_2_42DPEAAXX	8662582<br>8943791	649693650<br>670784325
2	NMD-deficient: RNAi against UPF2	51	rU2	single-end<br>single-end	75<br>75	AGC_BOSS_3_42DPEAAXX<br>AGC_BOSS_4_42DPEAAXX	8957760<br>8986487	671832000<br>673986525
Sample_2011_0059	Control: no RNAi (cells fed with Klebsiella)	51	tK2	paired-end	101	2011_0059_ACAGTG_L001_R1_001<br>2011_0059_ACAGTG_L001_R2_001	32147503	6493795606
Sample_2011_0055	Control: no RNAi (cells fed with Klebsiella)	51	tK3	paired-end	101	2011_0055_TGACCA_L001_R1_001<br>2011_0055_TGACCA_L001_R2_001	32103515	6484910030
Sample_2011_0060	NMD-deficient: RNAi against UPF3	51	rU3	paired-end	101	2011_0060_TTAGGC_L003_R1_001<br>2011_0060_TTAGGC_L003_R2_001	27414510	5537731020
Sample_2011_0056	NMD-deficient: somatic deletion of UPF1A	51	dA	paired-end	101	2011_0056_CAGATC_L001_R1_001<br>2011_0056_CAGATC_L001_R2_001	32072473	6478639546
Sample_2011_0057	NMD-deficient: somatic deletion of UPF1B	51	dB	paired-end	101	2011_0057_GATCAG_L003_R1_001<br>2011_0057_GATCAG_L003_R2_001	26085452	5269261304
Sample_2011_0058	NMD-deficient: somatic deletion of UPF1A and UPF1B	51	dAB	paired-end	101	2011_0058_CGTACG_L003_R1_001<br>2011_0058_CGTACG_L003_R2_001	25645448	5180380496

Supplemental Table S2: Number of introns or cryptic introns showing evidence of alternative splicing in RNAseq samples from WT or NMD-deficient paramecia.

	WT cells	NMD-deficient cells	All data
Number RNAseq reads (x 1e6)	165.1 M	258.0 M	423.1 M
Number (%) of introns with at least one IR event detected	40,363 (61.9%)	49,143 (75.4%)	53,716 (82.4%)
Number (%) of introns with at least one ASSV event detected	5,556 (8.5%)	14,587 (22.4%)	15,494 (23.8%)
Number of cryptic introns with at least one splicing event detected	5,151	19,146	20,719

Supplemental Table S3: RNAseq libraries analyzed to quantify ASSV in human.

Sample_ID	Accession	Tissue
Adipose_Breast_R1	ERR030880	Adipose
Adipose_Breast_R2	ERR030888	Adipose
Adipose_Breast_R3	ERR030883	Adipose
Adipose_Breast_R4	ERR030891	Adipose
Adrenal_R1	ERR030881	Adrenal
Adrenal_R2	ERR030889	Adrenal
Amnion_R1	SRR635193	Amnion
Amnion_R2	SRR638932	Amnion
Brain_R1	ERR030882	Brain
Brain_R2	ERR030890	Brain
hsa_br_F1	SRR306838	Brain
hsa_br_M1	SRR306840	Brain
hsa_br_M2	SRR306841	Brain
hsa_br_M3	SRR306839	Brain
hsa_br_M4	SRR306842	Brain
hsa_br_M5	SRR306843	Brain
FrontalGyrus_old_R1	SRR090441	Brain_FrontalGyrus
FrontalGyrus_old_R2	SRR090442	Brain_FrontalGyrus
FrontalGyrus_old_R3	SRR111901	Brain_FrontalGyrus
FrontalGyrus_old_R4	SRR112672	Brain_FrontalGyrus
FrontalGyrus_young_R2	SRR107727	Brain_FrontalGyrus
FrontalGyrus_young_R3	SRR112600	Brain_FrontalGyrus
FrontalGyrus_young_R4	SRR111895	Brain_FrontalGyrus
FrontalGyrus_young_R5	SRR111896	Brain_FrontalGyrus
FrontalGyrus_young_R6	SRR111897	Brain_FrontalGyrus
FrontalGyrus_young_R7	SRR111898	Brain_FrontalGyrus
FrontalGyrus_young_R8	SRR111899	Brain_FrontalGyrus
FrontalGyrus_young_R9	SRR111900	Brain_FrontalGyrus
Brain_STG_R1	ERR103421	Brain_STG
Brain_STG_R2	ERR103425	Brain_STG
Brain_STG_R3	ERR103426	Brain_STG
Brain_STG_R4	ERR103427	Brain_STG
Brain_STG_R5	ERR103428	Brain_STG
Brain_STG_R6	ERR103429	Brain_STG
Cerebellum_R4	SRR111935	Cerebellum
Cerebellum_R5	SRR111936	Cerebellum
Cerebellum_R6	SRR111937	Cerebellum
Cerebellum_R7	SRR112601	Cerebellum
Cerebellum_R8	SRR112673	Cerebellum
Cerebellum_R9	SRR112675	Cerebellum

hsa_cb_F1	SRR306844	Cerebellum
hsa_cb_M1	SRR306846	Cerebellum
Chorion_R1	SRR638936	Chorion
Chorion_R2	SRR638941	Chorion
Colon_R1	ERR030884	Colon
Colon_R2	ERR030892	Colon
Decidua_R1	SRR638937	Decidua
Decidua_R2	SRR638939	Decidua
EndomStromalCells_R1	SRR309129	Endometrial stromal cells
EndomStromalCells_R2	SRR309128	Endometrial stromal cells
ESC_H1_a_R1	SRR065492	ESC
ESC_H1_a_R2	SRR065493	ESC
ESC_H1_a_R3	SRR065504	ESC
ESC_H1_a_R4	SRR065526	ESC
ESC_H1_a_R5	SRR066678	ESC
ESC_H1_b_R1	SRR031628	ESC
Fibroblast_a_R1	SRR309267	Fibroblasts
Fibroblast_a_R2	SRR309268	Fibroblasts
Fibroblast_a_R3	SRR309269	Fibroblasts
Fibroblast_a_R4	SRR309270	Fibroblasts
Heart_R1	ERR030886	Heart
Heart_R2	ERR030894	Heart
hsa_ht_F1	SRR306847	Heart
hsa_ht_M1	SRR306848	Heart
hsa_ht_M2	SRR306850	Heart
Kidney_R1	ERR030885	Kidney
Kidney_R2	ERR030893	Kidney
hsa_kd_F1	SRR306851	Kidney
hsa_kd_M1	SRR306852	Kidney
hsa_kd_M2	SRR306853	Kidney
Liver_R1	ERR030887	Liver
Liver_R10	SRR087764	Liver
Liver_R11	SRR087765	Liver
Liver_R2	ERR030895	Liver
Liver_R6	SRR087756	Liver
Liver_R7	SRR087757	Liver
Liver_R8	SRR087758	Liver
Liver_R9	SRR087763	Liver
hsa_lv_M1	SRR306855	Liver
hsa_lv_M2	SRR306856	Liver
Lung_R1	ERR030879	Lung
Lung_R2	ERR030896	Lung

Lymph	ERR030878	Lymph
Muscle_R1	ERR030876	Muscle
Muscle_R2	ERR030899	Muscle
Muscle_b_R1	SRR087770	Muscle
Muscle_b_R10	SRR087779	Muscle
Muscle_b_R11	SRR087780	Muscle
Muscle_b_R12	SRR087781	Muscle
Muscle_b_R13	SRR094946	Muscle
Muscle_b_R14	SRR094947	Muscle
Muscle_b_R2	SRR087771	Muscle
Muscle_b_R3	SRR087772	Muscle
Muscle_b_R4	SRR087773	Muscle
Muscle_b_R5	SRR087774	Muscle
Muscle_b_R6	SRR087775	Muscle
Muscle_b_R7	SRR087776	Muscle
Muscle_b_R8	SRR087777	Muscle
Muscle_b_R9	SRR087778	Muscle
Ovary_R1	ERR030874	Ovary
Ovary_R2	ERR030901	Ovary
Placenta	SRR309266	Placenta
Prostate_R1	ERR030877	Prostate
Prostate_R2	ERR030898	Prostate
Testis_R1	ERR030873	Testis
hsa_ts_M1	SRR306857	Testis
hsa_ts_M2	SRR306858	Testis
Thyroid_R2	ERR030903	Thyroid
WhiteBloodCells_R1	ERR030875	WhiteBloodCells
WhiteBloodCells_R2	ERR030900	WhiteBloodCells
